# STUB1 downregulates TOP2A through a dual mechanism of ubiquitination and FOXM1-mediated transcription repression, suppressing breast cancer growth and enhancing sensitivity to chemotherapy

**DOI:** 10.1186/s11658-026-00902-2

**Published:** 2026-03-18

**Authors:** Baohui Yue, Qiaoling Xiang, Huimin Qiu, Mingxiang Huang, Mengxin Qi, Xianglan Yi, Sheng Zhou, Jing Xiong

**Affiliations:** Institute of Pathology, Tongji Hospital, Tongji Medical College, Huazhong University of Science and Technology, Wuhan, 430030 China

**Keywords:** Breast cancer, Chemosensitivity, STUB1, TOP2A, FOXM1

## Abstract

**Background:**

DNA topoisomerase IIɑ (TOP2A) is crucial for maintaining genomic stability and is an important target for genotoxic chemotherapeutic drugs. STIP1 homology and U-box-containing protein 1 (STUB1) is a U-box containing E3 ubiquitin ligase that participates in the degradation of specific oncogenic proteins. This research examined the potential regulatory function of STUB1 in relation to TOP2A, and explored its functional implications.

**Methods:**

To identify interactions between STUB1 and TOP2A, coimmunoprecipitation, Glutathione S-transferases (GST) pull-down, and immunofluorescence assays were performed. Cycloheximide (CHX) pulse-chase assay, in vivo and in vitro ubiquitination, quantitative RT-PCR, chromatin immunoprecipitation (CHIP), and luciferase assays were performed to determine how STUB1 interacts with TOP2A. In addition, TOP2A catalytic activity, colony formation, WST-1, and flow cytometry assays were performed and a xenograft model was further developed to explore whether STUB1 could downregulate the catalytic activity of TOP2A, reduce the growth of breast cancer, and increase its sensitivity to doxorubicin. Moreover, immunohistochemical staining was conducted to assess STUB1 and TOP2A expression levels, as well as their predictive roles in the efficacy of neoadjuvant chemotherapy in individuals diagnosed with breast cancer.

**Results:**

STUB1 enhanced TOP2A translocation to the cytoplasm, downregulating its expression through increased ubiquitination and degradation. Forkhead box M1 (FOXM1), another substrate of STUB1, served as a transcription factor for TOP2A, playing a role in STUB1-mediated downregulation of TOP2A at the transcriptional level. STUB1 inhibited TOP2A’s activity, reduced cancer cell proliferation, increased doxorubicin-induced apoptosis, and promoted cell cycle arrest. In a breast cancer xenograft model, STUB1 suppressed tumor growth and improved doxorubicin sensitivity. A positive correlation between FOXM1 and TOP2A expression was found in patients with breast cancer undergoing EC-T chemotherapy, both negatively correlated with STUB1, whose higher expression levels were linked to increased pathologic complete response (pCR) rates. STUB1 was evaluated as an independent predictor of pCR through univariate and multivariate analyses.

**Conclusions:**

This study proposes a novel function of STUB1 in the downregulation of TOP2A, which directly enhances sensitivity to chemotherapy.

**Supplementary Information:**

The online version contains supplementary material available at 10.1186/s11658-026-00902-2.

## Introduction

Breast cancer is the most common type of cancer among women worldwide. Advances in the discovery of therapeutic targets, as well as the development and application of new targeted drugs, have led to significant improvements in patient prognosis and an increase in 5-year survival rates. However, the heterogeneity of cancer cells, and the presence of mutations or other changes in therapeutic targets (such as hormone receptors and human epidermal growth factor receptor 2 [HER2]) as the disease progresses pose significant challenges to current treatment strategies. Therefore, novel targeted therapies are required for some primary breast cancers that currently lack therapeutic targets and for advanced, refractory, and metastatic breast cancer. Traditional genotoxic anticancer drugs, including anthracycline and platinum compounds, have been extensively utilized in the treatment of patients who are not suitable for hormonal or HER2-targeted therapy, and of patients with advanced or metastatic breast cancer. Genotoxic drugs are often administered as adjuvant therapies, especially to prevent postoperative recurrence. However, drug resistance often occurs, which can lead to poor treatment efficacy and cancer recurrence. The response of cancer cells to DNA damage is a key determinant of the efficacy of genotoxic drugs. This response includes initiating repair after cellular DNA damage, activation of cell-cycle checkpoints, and triggering cell apoptosis or aging, which ultimately determines cell fate and sensitivity to chemotherapy. Dysregulation of the DNA damage response may lead to resistance to these anticancer drugs.

DNA topoisomerase II (TOP2) is an important target of genotoxic drugs. It is an essential and widely present enzyme in eukaryotic cells that can alter the topological state of DNA, producing instantaneous double-strand breaks in entangled DNA fragments followed by reconnection in order to untangle DNA knots [1]. Some genotoxic drugs interfere with DNA breakage and reconnection by embedding into the binding site between TOP2 and the DNA double strand [2]. There are two isomers of TOP2 (TOP2A and TOP2B). TOP2B is predominantly expressed in quiescent cells, whereas TOP2A typically shows high expression in rapidly proliferating and actively growing cells. TOP2A exhibits elevated expression levels in various malignant tumors, including breast cancer, and is closely associated with abnormal proliferation of tumor cells, chromosomal aneuploidy, invasive phenotype, disease progression, tumor recurrence, and reduced overall survival [3]. TOP2A plays a crucial role in maintaining genomic stability [4], which is essential for normal cell growth and survival. Cancer cells depend on TOP2A because DNA stability is the basis for their continued proliferation and invasion. Moreover, it serves as a key factor to prevent DNA damage caused by chemotherapeutic drugs.

We found that the expression and effects of forkhead box M1 (FOXM1) and TOP2A are closely related in breast cancer tissues and cells. FOXM1 is a transcription factor that belongs to the forkhead box (FOX) family and plays a pivotal role in many biological processes, including cell cycle regulation, DNA damage repair, cell aging, and tissue development [5–7]. FOXM1 is also associated with many disease processes, especially tumors, owing to its unique role in the regulation of the cell cycle. As an oncogenic transcription factor, FOXM1 activates a network of genes involved in tumorigenesis and cancer progression [8]. Recent studies have also shown that, similar to TOP2A, FOXM1 is essential in regulating the DNA damage response and the sensitivity to genotoxic chemotherapy drugs [9], but the specific mechanism involved in FOXM1 dysregulation and its possible relationship with TOP2A remain unclear.

STIP1 homology and U-box-containing protein 1 (STUB1), also known as the carboxyl terminus of Hsc70-interacting protein (CHIP), possesses chaperone activity as well as U-box-dependent E3 ubiquitin ligase activity [10]. It participates in multiple biological functions in cells, including protein folding and degradation. STUB1 can bind to heat shock proteins (Hsp70 and Hsp90, for instance) and participate in the folding of immature or damaged proteins. When the substrate protein cannot fold correctly, STUB1 labels the substrate protein with ubiquitin, which is normally followed by degradation through the proteasome pathway. In neurodegenerative diseases, studies have found that STUB1 participates in the clearance and stability regulation of abnormal proteins [11, 12]. In addition, STUB1 is also involved in the occurrence and progression of tumors. STUB1 functions as a tumor suppressor by facilitating the ubiquitination and subsequent degradation of various oncogenic proteins, including mutant p53 [13], c-Myc [14], and ErbB2/HER2 [15]. In this study, FOXM1 and TOP2A were identified as novel substrates ubiquitinated by STUB1. We found that STUB1 reduced the expression of FOXM1 and TOP2A at the post-translational level. Furthermore, FOXM1 acted as a transcription factor for TOP2A, and STUB1 also regulated TOP2A expression at the transcriptional level through FOXM1. Therefore, STUB1 suppressed breast cancer growth and increased sensitivity to doxorubicin in vitro and in vivo. Moreover, the correlation between the expression of STUB1, FOXM1, and TOP2A and their ability to predict the efficacy of neoadjuvant chemotherapy were validated in patients with breast cancer. This study proposes an important regulatory mechanism involving STUB1, FOXM1, and TOP2A, which directly impacts the growth of tumor cells as well as their responsiveness to chemotherapy, demonstrating significant clinical potential.

## Methods

### Cell culture and reagents

A total of six breast cancer cell lines were used in this study, including SUM159 (Provider: Wuhan Pricella Biotechnology Company [CL-0622]), MCF-7 (Cat. No. HTB-22, Provider: American Type Culture Collection [ATCC]), ZR-75–1 (Cat. No. CRL-1500, Provider: ATCC), MDA-MB-231 (Cat. No. HTB-26, Provider: ATCC), MDA-MB-468 (Cat. No. HTB-132, Provider: ATCC), and BT474 (Cat. No. HTB-20, Provider: ATCC). All cell lines were routinely identified through short tandem repeat (STR) analysis and confirmed to be free of mycoplasma contamination. Doxorubicin, cycloheximide (CHX), MG132, and actinomycin D were purchased from MedChemExpress (MCE), diluted to the appropriate concentration before use, and stored according to the manufacturer’s instructions.

### Plasmids and transfection

Vector expressing various truncated and mutant versions of human STUB1, FOXM1, and TOP2A were constructed. Specifically, we constructed Myc-STUB1, Flag-FOXM1, and Flag-TOP2A. The GST-tagged version of STUB1 and Flag-tagged version of TOP2A were constructed for the glutathione S-transferase (GST) pull-down assay. STUB1 mutants included STUB1 K30A, STUB1 T246M, and STUB1 K260Q. The siSTUB1 sequence was 5’-CTGTGAAGGCGCACTTCTT-3’ and 5’-AGCGCTGGAACAGCATTGA-3’. Transfections were performed using Lipofectamine 3000 reagent (Invitrogen). Overexpression and knockdown efficiencies were assessed using western blotting.

### Western blot

The expression levels of STUB1, FOXM1, TOP2A, β-Tubulin, GST, Myc, Flag, and ubiquitin were assessed by immunoblotting using the following antibodies: anti-STUB1 (ab134064, Abcam), anti-FOXM1 (ab245309, Abcam), anti-FOXM1 (13147–1-AP, Proteintech), anti-TOP2A (A16440, ABclonal), anti-TOP2A (24641–1-AP, Proteintech), anti-β-Tubulin (ABL1030, Abbkine), anti-GST (ab138491, Abcam), anti-Myc (sc-40, Santa Cruz), anti-Flag (20543–1-AP, Proteintech), and anti-ubiquitin (A19686, ABclonal).

### Co-immunoprecipitation

At 48 h after transfection with the relevant vector, MCF-7 cells were lysed and centrifuged at 4 °C and at 12,000 rpm for 15 min. The resulting cell lysate was incubated with the corresponding antibody overnight at 4 °C on a rotator. On the second day, magnetic beads were added to combine with the antigen–antibody complexes. After 2 h, the beads were washed three times with a washing buffer. Proteins were denatured with 1× loading buffer and analyzed through immunoblotting.

### GST pull-down assay

GST-tagged STUB1 and truncated versions of it were expressed in an *Escherichia coli* expression system. Flag-tagged TOP2A and the relevant fragment plasmids were expressed in an HEK293 expression system. Equal amounts of GST-STUB1 and Flag-TOP2A or their truncated versions were mixed in the binding buffer. After addition of the relevant antibody, the mix was rotated at 4 °C for 12 h. Protein A/G magnetic beads (B23202, Selleck) were then added, and the sample was incubated for 2 h. The beads were then washed three times, and 1× loading buffer was used to denature the proteins, which were then analyzed using sodium dodecyl sulfate polyacrylamide gel electrophoresis (SDS-PAGE) and immunoblotting.

### Immunofluorescence

Cells were seeded in a confocal microplate, and transfected with the Myc-STUB1 construct or an empty vector. The cells were fixed with 4% paraformaldehyde for 15 min and permeabilized with 0.5% Triton X-100 for 10 min. The cells were then sequentially incubated with anti-Myc and anti-Flag antibodies, with fluorescein-coupled secondary antibodies, and with DAPI. A confocal microscope (Olympus SpinSR) was used to image the cells.

### CHX pulse-chase assay

The protein synthesis inhibitor CHX (50 μg/mL) was added to the culture medium at defined time points before cell lysis. The half-life of FOXM1 and TOP2A were detected using immunoblotting.

### *In vivo* ubiquitination assay

Sample cells were treated with MG132 (10 μM) for 8 h before collection. An anti-Flag antibody was added to enrich the corresponding antigenic complexes. Subsequently, ubiquitination was measured via western blotting using an anti-ubiquitin antibody.

### *In vitro* ubiquitination assay

The reaction system was as follows: 20 μL of a reaction system that included 1 μg E1, 1 μg E2, 2 μg ubiquitin, 2 μL 10 × Reaction Buffer, 5 μg Flag-TOP2A, and 5 μg GST-STUB1 were mixed and incubated in a metal bath at 37 °C for 90 min. The products were analyzed via western blotting.

### Quantitative RT-PCR (qPCR)

RNA was extracted from cells using Trizol (Invitrogen). ReverTra Ace qPCR RT Master Mix (TOYOBO, FSQ-201) was used for reverse transcription. The protocol was performed following the ChamQ Universal SYBR qPCR Master Mix (Q711-02, Vazyme) manual. RNA synthesis was terminated by adding actinomycin D to the medium to assess mRNA turnover. Subsequently, cells were collected at various time points, and mRNA expression level was measured using qPCR.

### Chromatin immunoprecipitation (CHIP)

Cells were fixed and crosslinked with formaldehyde. Excess formaldehyde was removed using glycine. The cells were then scraped and counted. An appropriate number of cells were lysed on ice via ultrasonication. The supernatant was immunoprecipitated with an anti-FOXM1 antibody. After washing and elution, crosslinks were reversed at 65 °C for 4 h. The results were analyzed via qPCR.

### Luciferase assay

Plasmids containing the wild type or mutant versions of the TOP2A promoter were separately transfected into 293 T cells. Equal amounts of lysate were added to the firefly luciferase substrate, and the fluorescence value was measured using a luminometer. The reaction was terminated using a stop reaction buffer. *Renilla* luciferase substrate was then added, and the fluorescence value was read immediately.

### TOP2A catalytic activity assay

The TOP2A catalytic activity assay was conducted using a human topoisomerase II assay kit (TG1001-1, TopoGEN). The lysate was used as the reaction enzyme, combined with kinetoplast DNA (kDNA) and reaction buffer, and incubated at 37 °C for 15 min. The reaction was then terminated, and substrate degradation was analyzed via DNA gel electrophoresis.

### Clonogenic assay, WST-1 assay, and flow cytometry

These assays were performed on four experimental subgroups: vehicle, STUB1, TOP2A, and STUB1 + TOP2A. For the clonogenic assays, 200 cells from each group were counted and seeded in 6-well plates. The cells were cultured for 2 weeks. After fixing with 4% paraformaldehyde and staining with 0.5% crystal violet, images were obtained and the number of colonies was determined. For the WST-1 assay, various groups of cells were seeded in 96-well plates and treated with doxorubicin. After 4 h of treatment with the WST-1 solution, the values were read using a microplate reader. Flow cytometry was used to detect apoptosis and cell-cycle progression in cancer cells. The cells were stained with FITC-annexin V and propidium iodide (PI) in the apoptosis assay. For the cell-cycle assay, the cells were fixed with 75% ethanol overnight and stained with PI/RNase staining buffer.

### Xenograft model

Specific pathogen-free (SPF) nude mice (BALB/c, approximately 18 g, 4 weeks old) were randomly divided into eight groups. Cells stably expressing proteins were subcutaneously injected into the right inguinal region of female mice (1.5 × 10^6^ cells in 150 μL PBS). Tumors were allowed to develop for 10 days, and doxorubicin or an equal volume of PBS was subsequently injected intraperitoneally (3 mg/kg, once per week during 2 weeks). After treatment, the experimental mice were humanely euthanized, and tumor tissues were collected for measurement and immunoblotting.

### Patients

A total of 115 patients with invasive breast cancer who had received neoadjuvant chemotherapy with EC-T at Tongji Hospital between 2020 and 2022 were included. The study of patients complies with the ethical guidelines of the 1975 Declaration of Helsinki and has been approved by the Ethics Committee of Tongji Hospital, Tongji Medical College, Huazhong University of Science and Technology (approval no.: TJ-IRB20221304, 23 December, 2022). Pathological complete response (pCR) was estimated in combination with preoperative fine-needle aspiration biopsy and postoperative pathology. H-score (0–300) evaluation and molecular subtyping were performed as previously described [16].

### Immunohistochemistry

After staining with DAB and hematoxylin, the slides were magnified, and the integrated option density (IOD) measured using Fiji. The average optical density (AOD) was calculated using the formula AOD = IOD/area.

### Statistical analysis

GraphPad Prism version 9.0 and SPSS Statistics version 27.0 were used for data analysis. The Pearson correlation coefficient was employed to determine the relationship between the expression levels of STUB1 and TOP2A. The two-tailed Student’s *t*-test was utilized to evaluate differences between two groups. The statistical results are expressed as the mean ± standard deviation (SD). *P < 0.05, **P < 0.01, and ***P < 0.001.

## Results

### TOP2A is negatively regulated by STUB1

**Fig. 1:**
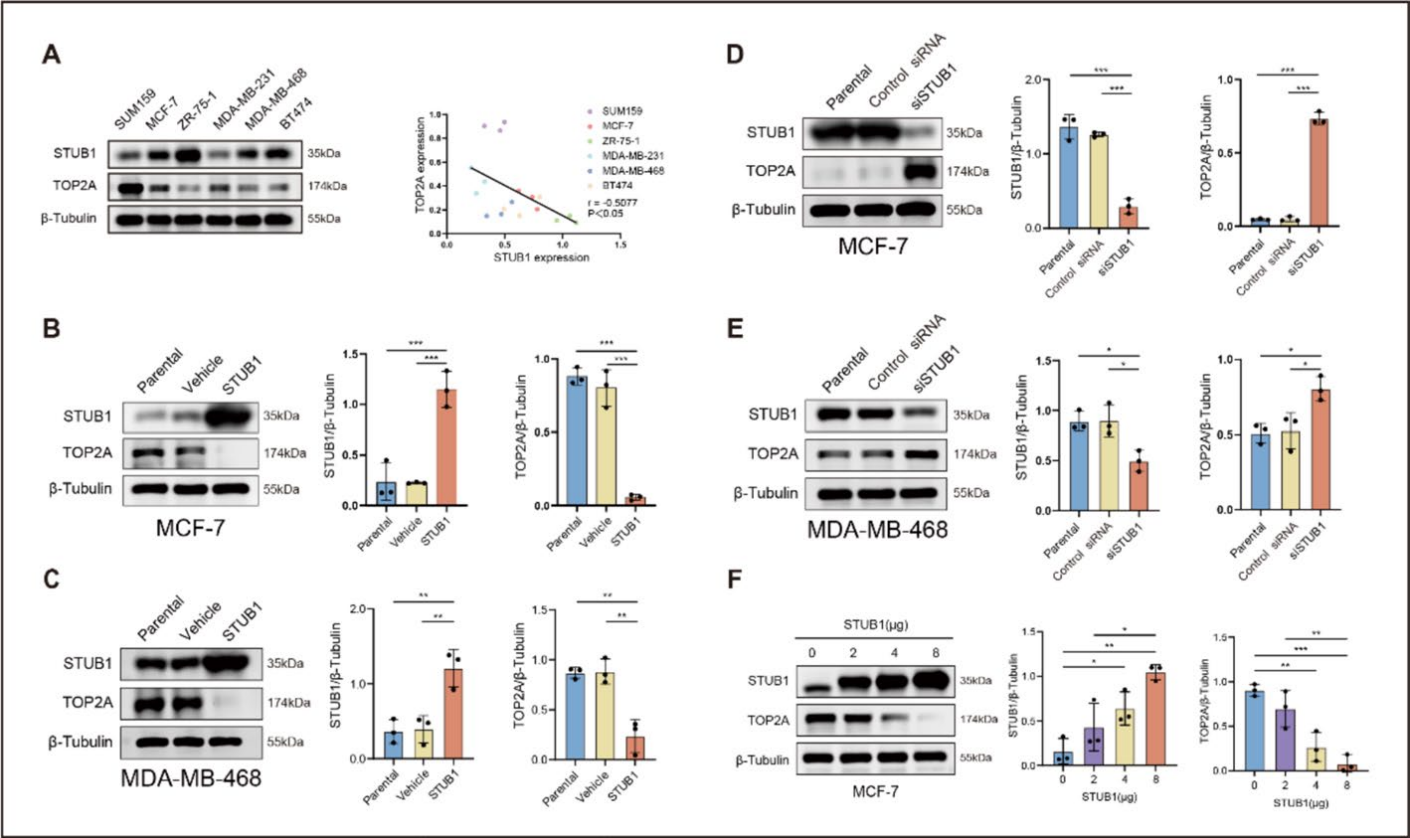
TOP2A is negatively regulated by STUB1. **A** Expression levels of STUB1 and TOP2A, and correlation between their relative quantities in several breast cancer cell lines. **B**, **C** TOP2A expression in MCF-7 and MDA-MB-468 cells following STUB1 transfection. **D**, **E** TOP2A expression in MCF-7 and MDA-MB-468 cells following siSTUB1 transfection. **F** Changes in TOP2A expression following increasing levels of STUB1 expression in MCF-7 cells. Histograms represent densitometric analyses of the STUB1 and TOP2A proteins. *P < 0.05, **P < 0.01, and ***P < 0.001

We investigated the expression levels of STUB1 and TOP2A in different human breast cancer cell lines and observed a negative correlation between STUB1 and TOP2A expression (*r* = −0.5077, *P* < 0.05). Specifically, the cell lines ZR-75–1 and BT474 demonstrated higher STUB1 levels alongside lower TOP2A levels. Conversely, the SUM159 and MDA-MB-231 cell lines exhibited lower STUB1 levels coupled with higher TOP2A levels (Fig. 1A).

To confirm this correlation, we performed STUB1 transfection or silencing in MCF-7 and MDA-MB-468 cells. Figure 1B, C showed that STUB1 transfection decreased the expression of TOP2A, whereas STUB1 silencing increased it (Fig. 1D, E). These results suggest that STUB1 negatively regulates TOP2A expression. Moreover, the negative regulation of TOP2A by STUB1 was dose-dependent, with increasing levels of transfected STUB1 leading to a gradual decrease in TOP2A expression (Fig. 1F).

### Binding between STUB1 and TOP2A *in vivo* and *in vitro*

**Fig. 2:**
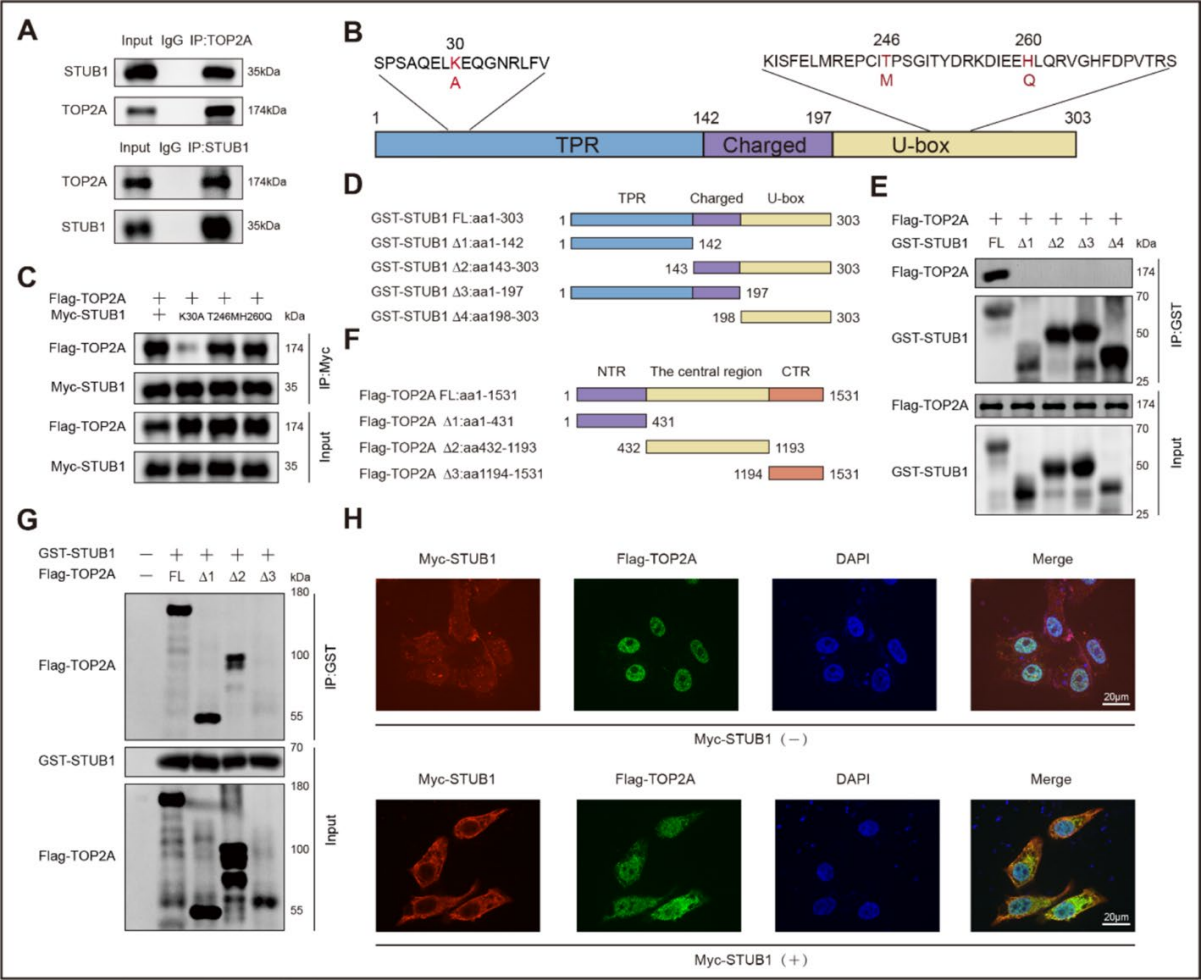
Binding between STUB1 and TOP2A in vivo and in vitro. **A** Binding between endogenous STUB1 and TOP2A detected by co-immunoprecipitation assay using antibodies against STUB1 or TOP2A in MCF-7 cells. **B** Diagram showing the STUB1 mutants K30A, T246M, and H260Q. **C** Wild-type STUB1 or mutant T246M and H260Q could interact with TOP2A, while mutant K30A could not. **D** Diagram showing the different structural domains of STUB1 and the truncations used for the assays. **E** Binding between Flag-TOP2A and full-length GST-STUB1 or truncated versions of it, tested via GST pull-down assay. **F** Diagram showing the different structural domains of TOP2A and the truncations used for the assays. **G** Binding between GST-STUB1 and full-length Flag-TOP2A or truncated versions of it, tested via GST pull-down assay. (H) Confocal analysis of STUB1 and TOP2A subcellular localization. Scale bar, 20 μm

We then investigated the interaction between STUB1 and TOP2A. Co-immunoprecipitation and western blot assays showed that endogenous STUB1 bound to endogenous TOP2A (Fig. 2A) in a chaperone-dependent manner. STUB1 K30A, a mutant that lacks the ability to interact with molecular chaperones [17] (Fig. 2B), failed to bind to TOP2A (Fig. 2C). The binding of STUB1 to TOP2A is independent of the E3 ubiquitin ligase activity of STUB1. The mutants T246M and H260Q, which eliminate the E3 ubiquitin ligase activity of STUB1 [17–19] (Fig. 2B), retained their capacity to bind TOP2A (Fig. 2C).

To further identify the specific domains of STUB1 and TOP2A that participate in the binding, we developed several truncated versions of each protein and conducted a GST pull-down assay. STUB1 contains 303 amino acids (aa) and comprises three structural domains: the TPR domain, the U-box domain, and the intervening region between the TPR and U-box domains. TPR is the N-terminus domain that interacts with the molecular chaperones Hsc70‐Hsp70 and Hsp90. U-box is the C-terminus domain, with the E3 ubiquitin ligase activity of STUB1 is restricted to it [17]. For the present study, four STUB1 truncations were constructed: STUB1Δ1 (aa 1–142), STUB1Δ2 (aa 143–303), STUB1Δ3 (aa 1–197), and STUB1Δ4 (aa 198–303) (Fig. 2D). The GST pull-down assay revealed that only the full-length STUB1 interacted with TOP2A. All the truncated versions of STUB1 failed to bind to TOP2A (Fig. 2E).

TOP2A has three functional domains: an N-terminus with ATPase activity, a central region involved in DNA interaction and cleavage, and a C-terminus that regulates cell proliferation [20]. Correspondingly, three truncated versions of TOP2A were generated: TOP2AΔ1 (aa 1–431), TOP2AΔ2 (aa 432–1193), and TOP2AΔ3 (aa 1194–1531) (Fig. 2F). Results from the GST pull-down assays indicated that STUB1 interacted with the N-terminus and central region of TOP2A. No binding was detected between STUB1 and the C-terminus of TOP2A (Fig. 2G).

In addition, immunofluorescence experiments were performed to observe the subcellular localization of STUB1 and TOP2A. As shown by the distribution of green fluorescence, Flag-TOP2A was mainly expressed in the nucleus, whereas Myc-STUB1 (indicated by red fluorescence) was mainly localized to the cytoplasm. Myc-STUB1 transfection facilitated the translocation of Flag-TOP2A from the nucleus to the cytoplasm, where it interacted with Myc-STUB1. Digital merging of the individual images showed significant colocalization of these two proteins in the cytoplasm, as indicated by the presence of yellow fluorescence (PCC = 0.80) (Fig. 2H).

### STUB1 promotes TOP2A ubiquitination and degradation

**Fig. 3:**
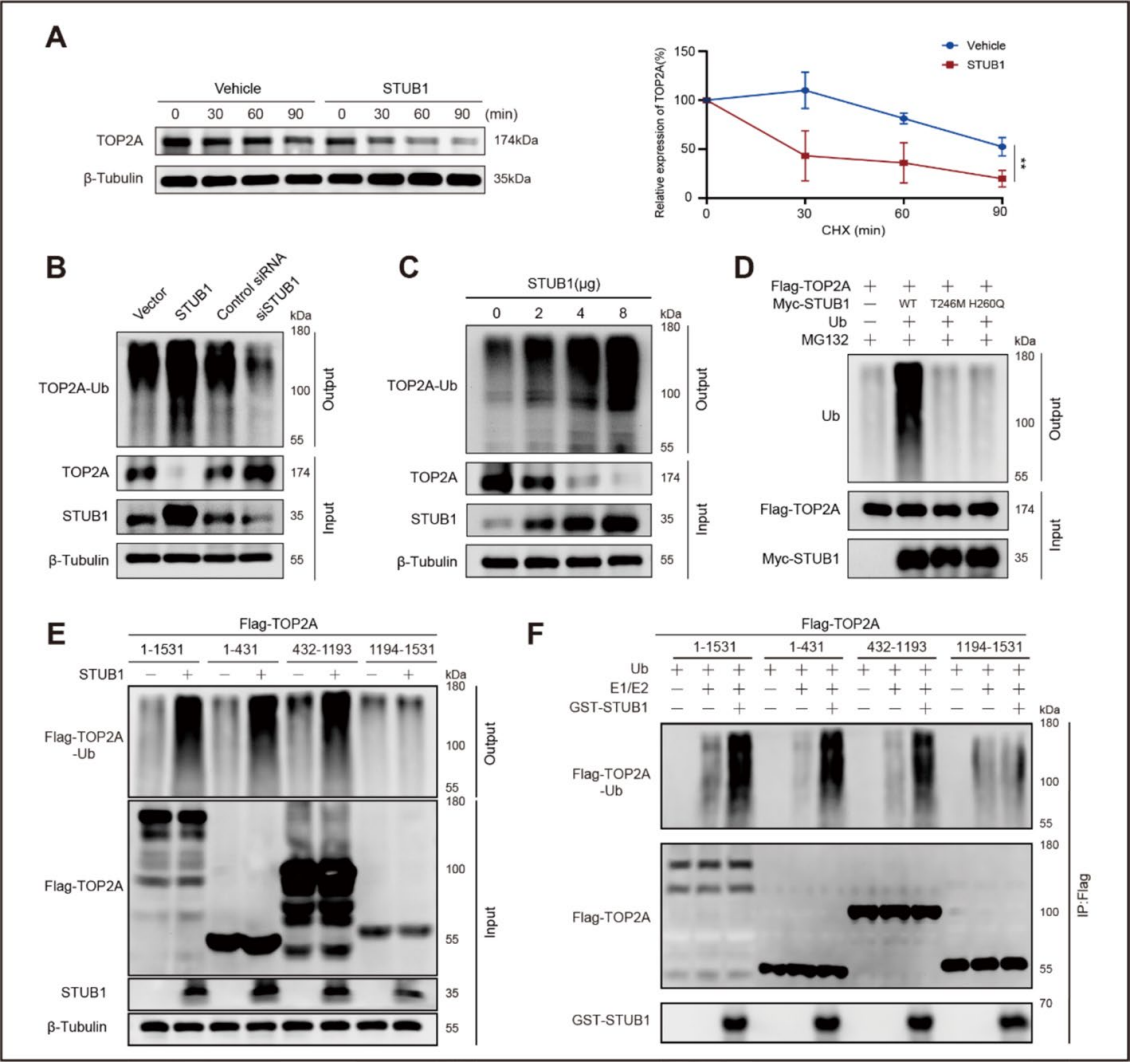
STUB1 downregulates TOP2A through the mechanism of ubiquitination. **A** Degradation of TOP2A in MCF-7 cells following transfection with a STUB1-containing vector or the corresponding empty vector after CHX treatment. The relative expression profiles of TOP2A are shown. Data are presented as mean ± SD from three independent experiments. **P < 0.01. **B** Ubiquitination of TOP2A following transfection with STUB1 or siSTUB1. **C** Dose–response ubiquitination of TOP2A after transfection with a STUB1-containing vector. **D** Wild-type STUB1 but not the T246M and H260Q mutants increased the ubiquitination of TOP2A. **E** In vivo ubiquitination assay demonstrating the ubiquitination levels of full-length TOP2A aa 1–1531 and its fragments aa 1–431, aa 432–1193 and aa 1194–1531 under effects of STUB1. **F** In vitro ubiquitination assay demonstrating the ubiquitination levels of full-length TOP2A and its fragments under effects of GST-STUB1

Given that STUB1 is an E3 ubiquitin ligase, we hypothesized that STUB1 may induce the ubiquitination and degradation of TOP2A. A CHX pulse-chase assay was conducted to determine the half-life of TOP2A. The findings indicated that STUB1 induced TOP2A degradation at the post-translational level. In control cells, the half-life of TOP2A was 94 min, whereas in STUB1-transfected cells, it was significantly reduced to 23 min (Fig. 3A).

Furthermore, in vivo ubiquitination assays demonstrated that STUB1 induces TOP2A ubiquitination. STUB1 transfection increased TOP2A ubiquitination, whereas STUB1 silencing decreased it (Fig. 3B). Moreover, STUB1 promoted TOP2A ubiquitination in a dose-dependent manner. The increase of STUB1 caused a gradual increase in TOP2A ubiquitination and a corresponding decrease in TOP2A expression (Fig. 3C).

It is known that the T246M and H260Q mutations eliminate the E3 ubiquitin ligase activity of STUB1 [17–19]. Compared with wild-type STUB1, the T246M and H260Q mutants failed to increase TOP2A ubiquitination (Fig. 3D). These results confirm that STUB1 functions as an E3 ligase for TOP2A, and its enzymatic activity is essential for TOP2A ubiquitination.

In addition, we tested whether binding to TOP2A was required for STUB1 to induce TOP2A ubiquitination. MCF-7 cells were co-transfected with His-Ub, STUB1, and Flag-tagged full-length TOP2A (aa 1–1531) or different Flag-tagged truncated variants (aa 1–431, aa 432–1193, and aa 1194–1531). An in vivo ubiquitination assay revealed that STUB1 elevated the ubiquitination of full-length TOP2A (aa 1–1531), and the aa 1–431 and aa 432–1193 truncated versions. However, STUB1 did not increase the ubiquitination of the version containing only aa 1194–1531 (Fig. 3E). Similarly, an in vitro ubiquitination assay confirmed that GST-STUB1 ubiquitinated full-length TOP2A (aa 1–1531) and the aa 1–431 and aa 432–1193 truncated versions, but not that corresponding to aa 1194–1531 (Fig. 3F).

### STUB1 inhibits TOP2A transcription through FOXM1

**Fig. 4:**
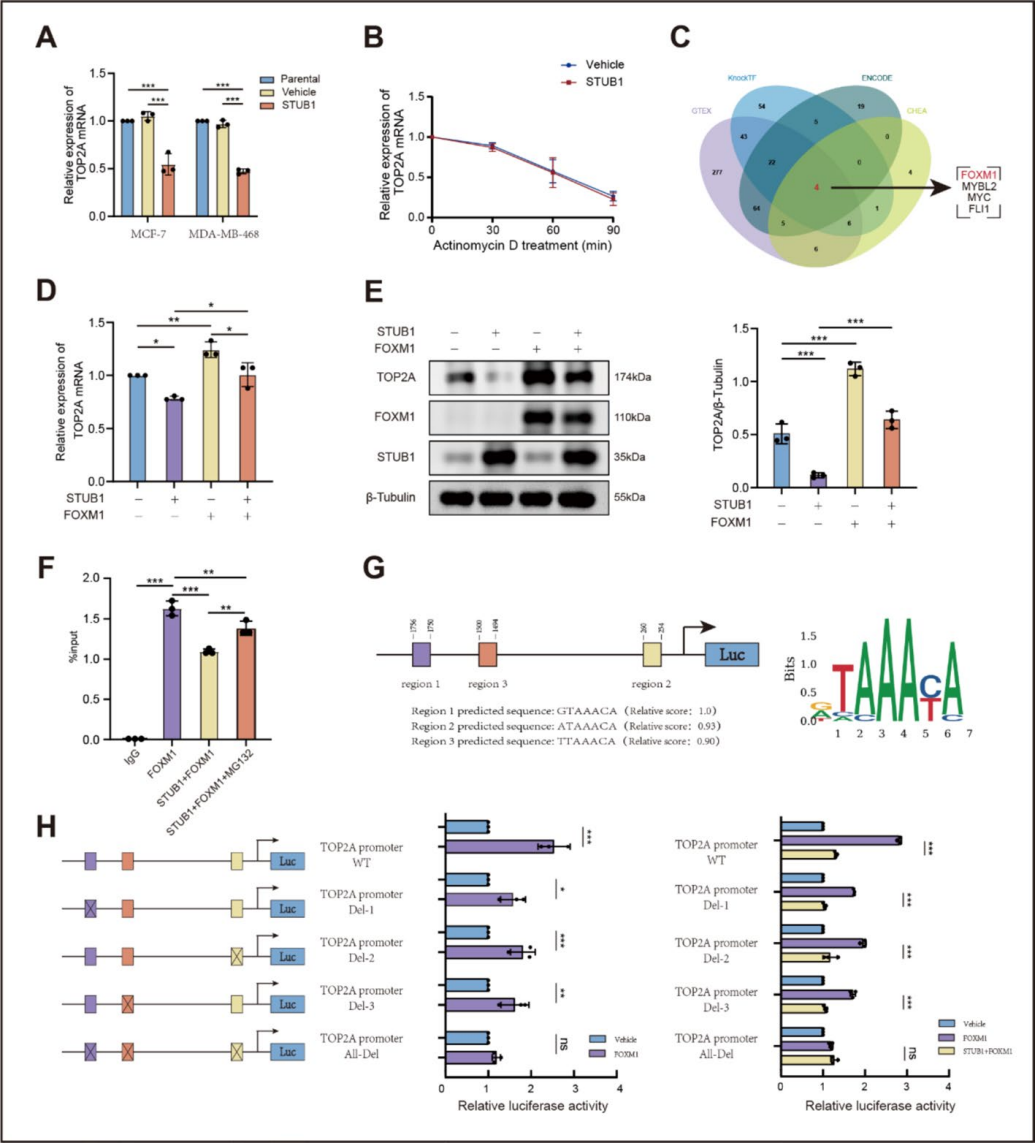
STUB1 inhibits TOP2A transcription through FOXM1. **A** mRNA expression of TOP2A in MCF-7 and MDA-MB-468 cells following transfection with a STUB1-containing vector, detected via qPCR. **B** Degradation of TOP2A mRNA in MCF-7 cells following treatment with actinomycin D. **C** Candidate transcription factors identified through bioinformatics analysis. **D**, **E** mRNA and protein levels of TOP2A in MCF-7 cells following transfection with a STUB1- or FOXM1-containing vector. **F** CHIP assay showing the binding between FOXM1 and the TOP2A promoter in MCF-7 cells. **G** Diagram showing the sequence and location of predicted binding sites of FOXM1 at the TOP2A promoter. **H** Luciferase assay showing the effect of FOXM1 and STUB1 on the activity of wild-type TOP2A promoter or TOP2A promoter mutants carrying deletions in one or all of the predicted binding sites: TOP2A promoter Del-1 (–1756 to –1750 bp deleted), TOP2A promoter Del-2 (–260 to –254 bp deleted), TOP2A promoter Del-3 (–1500 to –1494 bp deleted), and TOP2A promoter All-Del (all three regions deleted). Data are presented as mean ± SD from three independent experiments. *P < 0.05, **P < 0.01, and ***P < 0.001

Mechanistic studies revealed that mRNA levels of TOP2A also decreased after STUB1 transfection (Fig. 4A). An actinomycin D experiment was performed to determine whether this effect occurred at the post-transcriptional level. However, the half-life of TOP2A mRNA did not show any apparent changes (Fig. 4B). Therefore, we investigated whether STUB1 inhibited the promoter activity of TOP2A through a specific transcription factor. We interrogated four independent transcription factor databases-specifically, KnockTF (http://www.licpathway.net/KnockTF/), ENCODE (https://www.encodeproject.org/), GTEX (https://www.gtexportal.org/home%5B33), and CHEA (https://maayanlab.cloud/chea3/)-each constructed using distinct algorithms to identify potential upstream transcriptional regulators of TOP2A. After data analysis, FOXM1 was identified as a potential transcription factor (Fig. 4C). Preliminary verification using qPCR and western blot analysis revealed that FOXM1 increased the mRNA and corresponding protein levels of TOP2A. Conversely, STUB1 decreased the mRNA and corresponding protein levels of TOP2A in a FOXM1-dependent manner (Fig. 4D, E).

A CHIP assay revealed that FOXM1 bound to the TOP2A promoter. However, STUB1 decreased FOXM1 occupancy of the TOP2A promoter (Fig. 4F). Concurrently, we performed a rescue experiment by treating cells with proteasome inhibitor MG132. The results showed that MG132 treatment could partially restore the binding between FOXM1 and the TOP2A promoter that had been inhibited by STUB1. This suggests a potential influence of STUB1 on FOXM1; however, it is important to note that the inhibition of FOXM1’s binding to the TOP2A promoter by STUB1 is not solely mediated through STUB1-induced protein degradation.

Furthermore, a luciferase assay was conducted to confirm the precise binding location of FOXM1 on the TOP2A promoter and to determine whether STUB1 inhibited TOP2A transcription through FOXM1. The FOXM1 binding sites in the TOP2A promoter were predicted using JASPAR (https://jaspar.genereg.net/) (Fig. 4G). Three different versions of the TOP2A promoter with deletions on each one of these predicted binding sites (TOP2A promoter Del-1 [–1756 to –1750 bp], TOP2A promoter Del-2 [–260 to –254 bp], and TOP2A promoter Del-3 [–1500 to –1494 bp]), as well as a promoter with deletions in all three regions (All-Del) were synthesized and inserted into a luciferase-carrying vector. As shown in Fig. 4H, FOXM1 transfection enhanced luciferase activity in cells expressing the three different TOP2A promoter mutants, however, this was no longer the case in those expressing All-Del. This result confirmed that these three regions were binding sites. In addition, the luciferase assay showed that STUB1 inhibited the activity of the TOP2A promoter, since luciferase activity was significantly reduced upon STUB1 transfection. These findings suggest that STUB1 interferes with the interaction between FOXM1 and the TOP2A promoter, thereby suppressing TOP2A transcription.

### STUB1 is also an E3 ligase for FOXM1

**Fig. 5:**
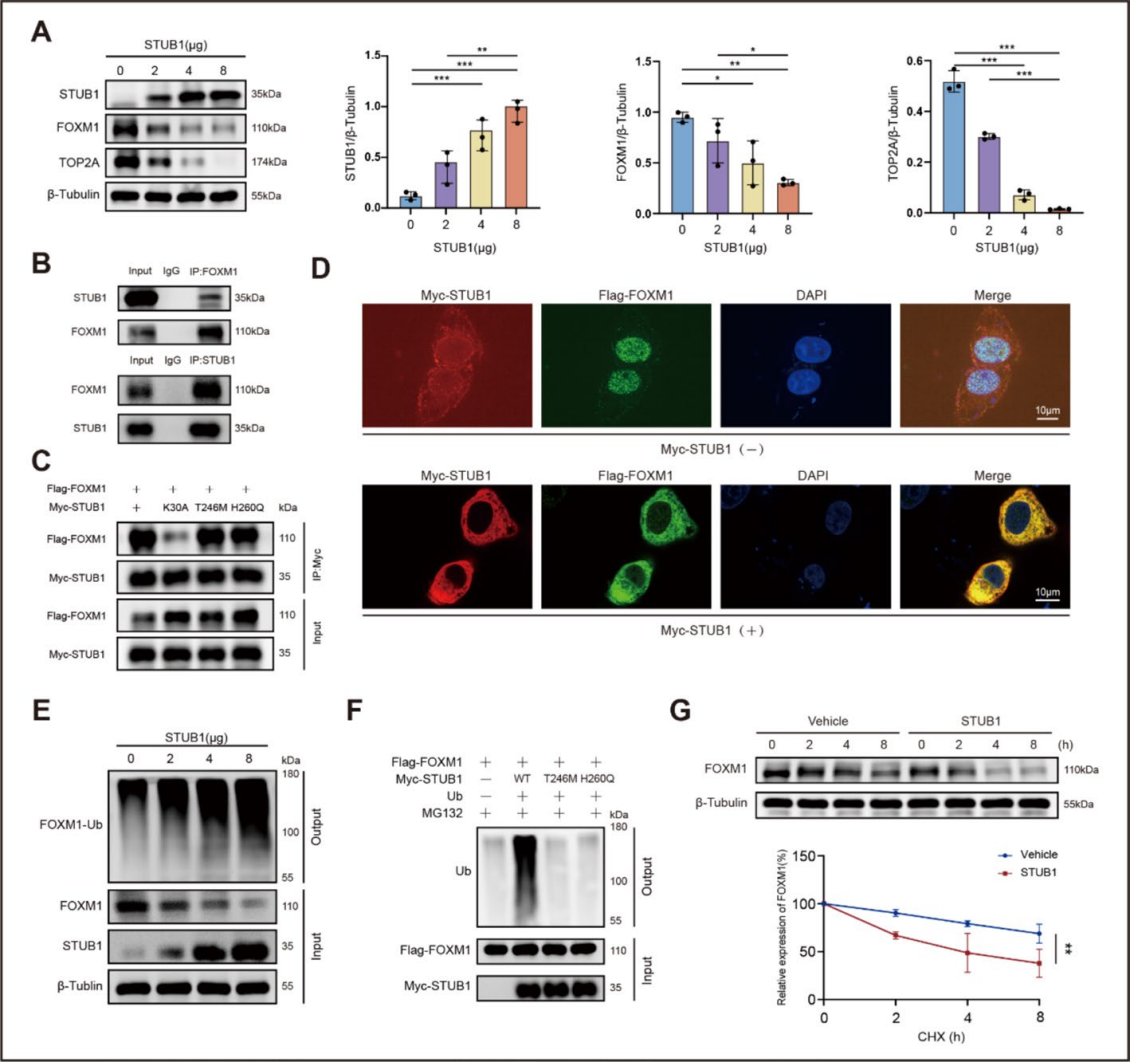
STUB1 is also an E3 ligase for FOXM1. **A** Dose-dependent decrease of FOXM1 and TOP2A expression after transfection with a STUB1-containing vector in MCF-7 cells. The histograms show the results of the densitometric analyses for each protein. **B** Binding between endogenous STUB1 and FOXM1 detected by co-immunoprecipitation assay using antibodies against STUB1 or FOXM1 in MCF-7 cells. **C** Wild-type STUB1 or mutant T246M and H260Q could interact with FOXM1, while mutant K30A could not. **D** Confocal analysis of STUB1 and FOXM1 subcellular localization. Scale bar, 10 μm. **E** In vivo ubiquitination assay showing dose–response ubiquitination of FOXM1 following transfection with a STUB1-containing vector. **F** Wild-type STUB1 but not the T246M or H260Q mutants increased FOXM1 ubiquitination. **G** Degradation of FOXM1 in MCF-7 cells with or without transfection of a STUB1-containing vector. Data are presented as mean ± SD from three independent experiments. *P < 0.05, **P < 0.01, and ***P < 0.001

Because STUB1 inhibits TOP2A transcription through FOXM1, we also examined the potential influence of STUB1 on FOXM1, as well as the underlying regulatory mechanisms involved. STUB1 exhibited a dose-dependent inhibitory effect on FOXM1 expression (Fig. 5A), and a significant interaction between STUB1 and FOXM1 was identified (Fig. 5B). This interaction was chaperone-dependent, since STUB1 K30A failed to interact with FOXM1 (Fig. 5C). Comparably, the mutants T246M and H260Q, which eliminate the E3 ubiquitin ligase activity of STUB1, retained their capacity to bind TOP2A. In addition, the immunofluorescence experiment revealed the translocation of Flag-FOXM1 (green signal) from the nucleus to the cytoplasm following transfection with Myc-STUB1 (red signal), reflecting a strong colocalization of FOXM1 with STUB1 in the cytoplasm (PCC = 0.97) (Fig. 5D).

Subsequently, an ubiquitination assay indicated that STUB1 was also an E3 ligase for FOXM1. STUB1 promoted FOXM1 ubiquitination in a dose-dependent manner (Fig. 5E). Likewise, the enzymatic activity of STUB1 was essential for FOXM1 ubiquitination. Compared with wild-type STUB1, both the T246M and H260Q mutants of STUB1 failed to increase the ubiquitination of FOXM1 (Fig. 5F). In agreement with these results, a CHX pulse-chase assay further showed that STUB1 induced FOXM1 degradation (Fig. 5G).

### STUB1 inhibits the catalytic activity of TOP2A and suppresses the growth of breast cancer cells, simultaneously increasing their susceptibility to doxorubicin

**Fig. 6:**
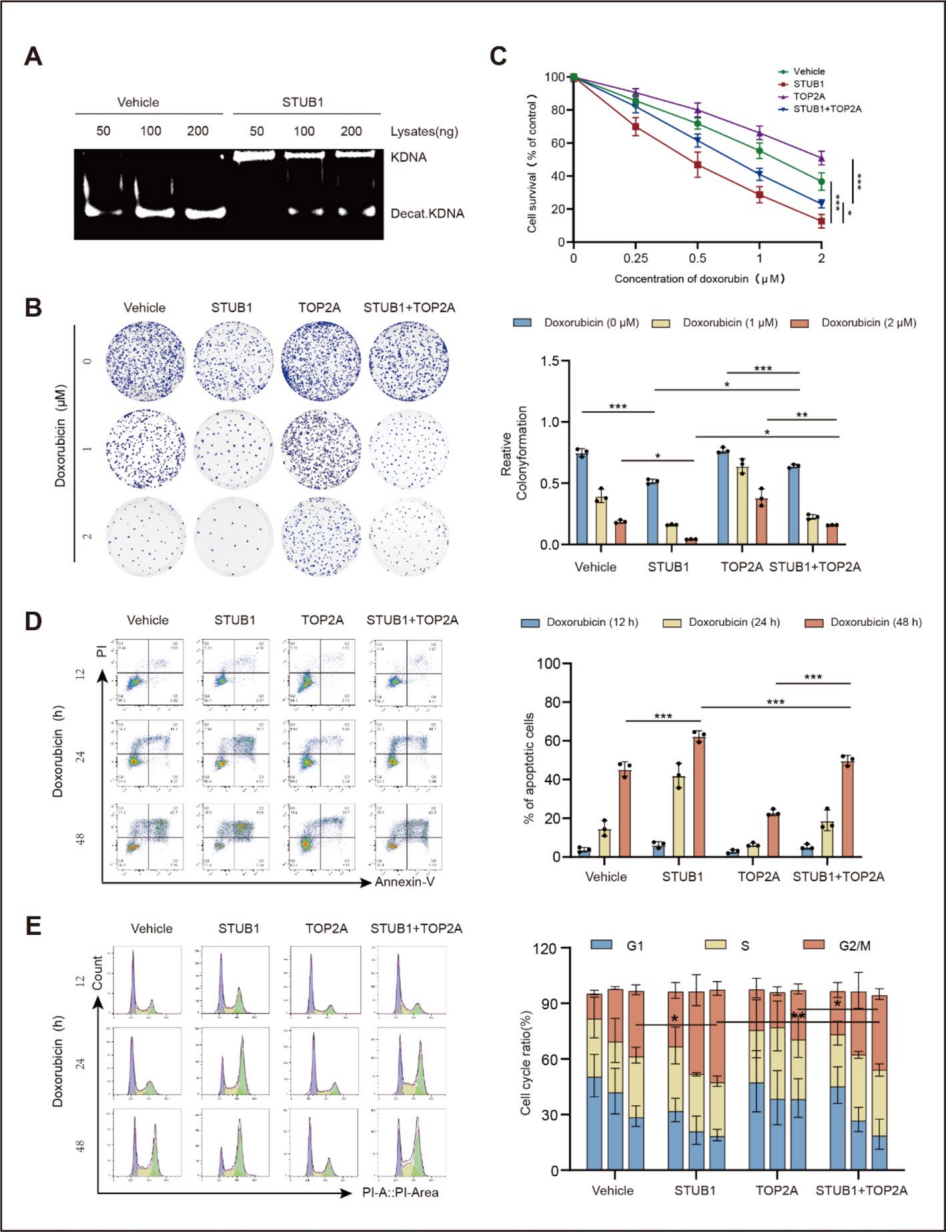
Effects of STUB1 on TOP2A catalytic activity, cancer cell growth, apoptosis, and cell cycle. **A** Kinetoplast DNA (kDNA) decatenation assay for the catalytic activity of TOP2A in MCF-7 cells. **B** Colony formation in MCF-7 cells transfected with vectors expressing STUB1, TOP2A, or STUB1 + TOP2A, assessed under different concentrations of doxorubicin. **C** WST-1 assay conducted to evaluate the viability of MCF-7 cells. **D** Apoptosis assay performed on MCF-7 cells transfected with vectors expressing STUB1, TOP2A, or STUB1 and TOP2A, following treatment with 1 μM doxorubicin for 12, 24, or 48 h. **E** Distribution of cell cycle phases in MCF-7 cells following doxorubicin treatment. *P < 0.05, **P < 0.01, and ***P < 0.001

Considering the negative effects of STUB1 on TOP2A expression, we investigated whether STUB1 could inhibit the catalytic activity of TOP2A, suppress the growth of cancer cells, and enhance their sensitivity to doxorubicin. The main function of TOP2A is to decatenate intertwined DNA. Therefore, its catalytic activity was accessed by detecting the decatenation of kinetoplast DNA mediated by TOP2A. The findings indicated that the catalytic activity of TOP2A was inhibited by STUB1. After transfection with STUB1, TOP2A-meditated decatenation of kinetoplast DNA significantly decreased (Fig. 6A).

Subsequently, colony formation, WST-1, and flow cytometry assays were performed using MCF-7 cells transfected with STUB1, TOP2A, or both, with or without doxorubicin treatment. In the presence or absence of doxorubicin, STUB1 suppressed colony formation by cancer cells (Fig. 6B). STUB1 also increased the responsiveness of cancer cells to the suppression of colony formation induced by doxorubicin (Fig. 6B), cytotoxicity (Fig. 6C), and apoptosis (Fig. 6D). In addition, STUB1 enhanced the arrest of the cell cycle in the G2/M phase induced by doxorubicin (Fig. 6E). However, these effects were less pronounced in cells co-transfected with TOP2A and STUB1 compared with those transfected with STUB1 alone (Fig. 6B–E). Therefore, the effect of STUB1 depends on TOP2A inhibition.

### STUB1 inhibits tumor growth and increases the response to doxorubicin in a breast cancer xenograft model

**Fig. 7:**
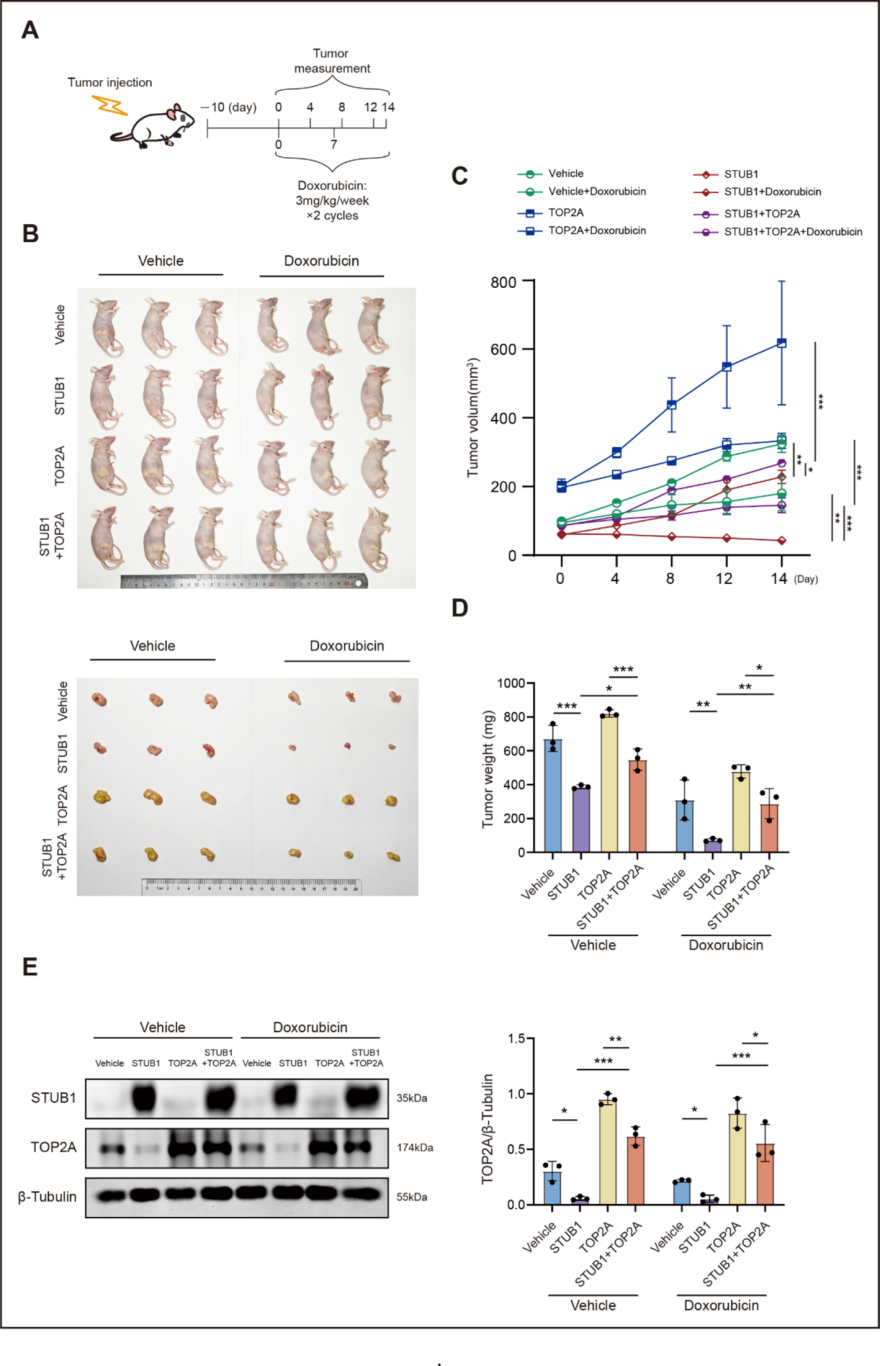
STUB1 inhibited the growth of xenograft tumors while simultaneously increasing their sensitivity to doxorubicin treatment. **A** Diagrammatic illustration of subcutaneous xenograft implantation process and schedule for doxorubicin administration. **B** Representative images of nude mice and tumors from each group. **C** Monitoring of xenograft size during the treatment period. **D** Tumor weight measured on day 14. **E** Levels of STUB1 and TOP2A proteins in xenograft tumor tissues assessed via western blot analysis. *P < 0.05, **P < 0.01, and ***P < 0.001

To further confirm in vitro observations, we established an in vivo xenograft model by implanting MCF-7 cells overexpressing STUB1, TOP2A, or STUB1 and TOP2A, followed by intraperitoneal administration of doxorubicin (3 mg/kg, once per week for 2 weeks) (Fig. 7A). The results demonstrated that STUB1 suppressed the xenograft tumor growth and enhanced its sensitivity to doxorubicin. With or without doxorubicin treatment, mice inoculated with cells previously transfected with STUB1 showed significant suppression of tumor growth compared with those inoculated with control cells. Similarly, the influence of STUB1 depended on its inhibitory effect on TOP2A. The decrease in tumor growth was less pronounced in mice co-transfected with TOP2A and STUB1 than in those inoculated with cells transfected with STUB1 alone (Fig. 7B–E).

### Correlations among the histological expression of STUB1, FOXM1, and TOP2A as predictive biomarkers for the efficacy of neoadjuvant chemotherapy in patients with breast cancer

**Fig. 8:**
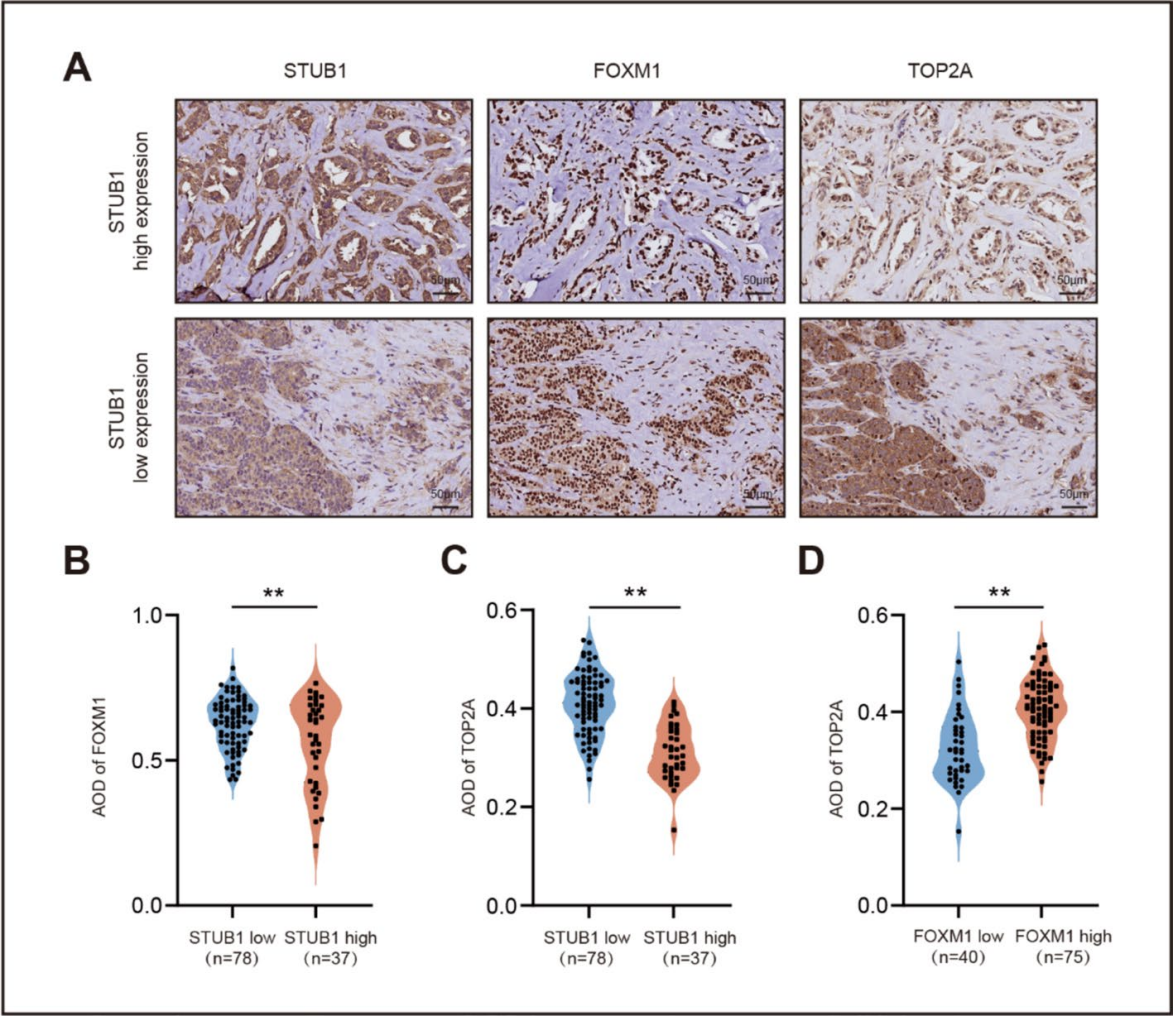
Correlations among the histological expression of STUB1, FOXM1, and TOP2A in breast cancer. **A** Immunohistochemical staining for STUB1, FOXM1, and TOP2A. Scale bar, 50 μm. **B** Correlations between STUB1 and FOXM1 expressions in patients. **C** Correlations between STUB1 and TOP2A expressions in patients. **D** Correlations between FOXM1 and TOP2A expressions in patients. **P < 0.01

To determine the clinical relevance of our research findings, we performed immunohistochemical staining to compare the levels of STUB1, FOXM1, and TOP2A in cancer tissues and their potential associations, as well as their relationship with the efficacy of chemotherapy in patients. Our study included 115 patients with breast cancer, all of whom underwent EC-T neoadjuvant chemotherapy. Representative images of immunohistochemical staining for STUB1, FOXM1, and TOP2A are shown in Fig. 8A. Quantification of the staining intensity showed a positive correlation between the expression of FOXM1 and TOP2A; however, both were negatively correlated with that of STUB1 (Fig. 8B–D).

In addition, we analyzed the correlation between STUB1, FOXM1, and TOP2A expressions, and chemotherapy efficacy. As shown in Table 1, response to chemotherapy significantly correlated with the expression levels of STUB1, FOXM1, and TOP2A. Patients with high STUB1 expression and low FOXM1 and TOP2A expression had significantly higher pCR rates. Univariate and multivariate analyses indicated that STUB1 and TOP2A were independent predictors of pCR (Table 2).

**Table 1:** Correlation between chemotherapy response and expression of STUB1, FOXM1, and TOP2A in invasive breast cancer

Subtypes	*n*	pCR rate (STUB1 high/low)	*P* value	pCR rate (FOXM1 high/low)	*P* value	pCR rate (TOP2A high/low)	*P* value
All patients	115	81.1%/41.0%	< 0.001	48.0%/65.0%	0.082	41.6%/78.9%	< 0.001
Luminal (non-HER2 +)	60	76.2%/56.4%	0.129	57.9%/72.7%	0.251	56.8%/73.9%	0.180
HER2 +	26	88.9%/29.4%	0.004	56.2%/40.0%	0.420	33.3%/87.5%	0.011
TNBC	29	85.7%/22.7%	0.003	23.8%/75.0%	0.011	22.7%/85.7%	0.003

**Table 2:** Univariate and multivariate analyses of pCR against various characteristics in patients with breast cancer who received neoadjuvant chemotherapy

Variable	pCR rate (%)	Univariate analysis *P* value	Multivariate analysis *P* value
STUB1 (high/low)	81.1%/41.0%	< 0.001	0.002
FOXM1 (high/low)	48.0%/65.0%	0.082	0.137
TOP2A (high/low)	41.6%/78.9%	< 0.001	0.015
Size (pT1-2/pT3-4)	53.0%/59.2%	0.511	0.302
Histological grade (I-II/III)	57.4%/48.9%	0.373	0.126
LN metastasis (posi/neg)	56.9%/51.6%	0.571	0.326
MIB (< 10%/ ≥ 10%)	68.0%/43.1%	0.008	0.057
ERα (posi/neg)	64.3%/37.8%	0.005	0.028
PR (posi/neg)	58.0%/47.8%	0.285	0.089
HER2 (posi/neg)	34.6%/59.6%	0.025	0.073
Molecular subtype (TNBC/others)	37.9%/59.3%	0.045	0.011

## Discussion

**Fig. 9:**
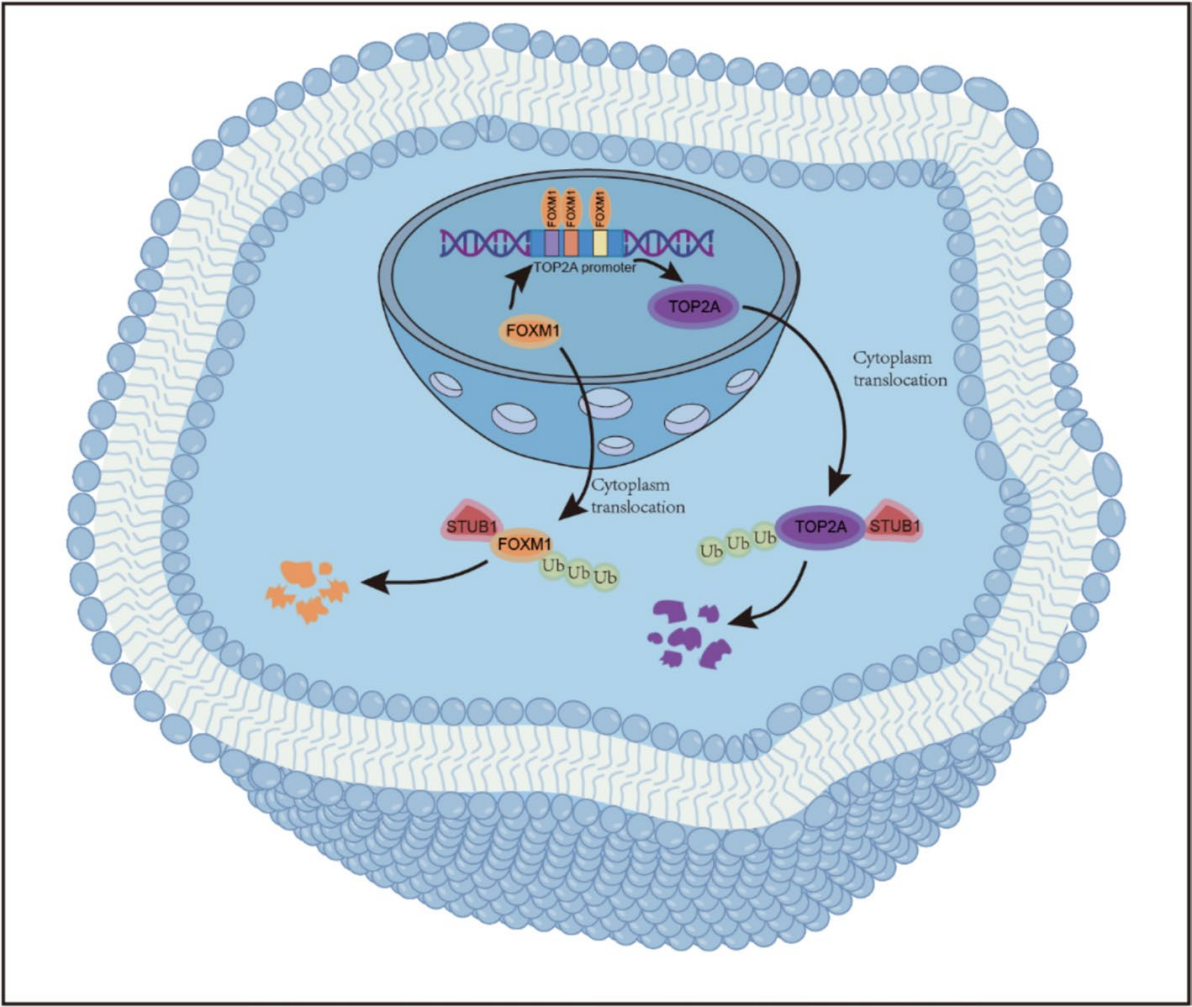
Proposed model for the regulation of TOP2A by STUB1. TOP2A is translocated from the nucleus to the cytoplasm through the action of STUB1, which directly binds to TOP2A and downregulates its levels at the post-translational level by promoting its ubiquitination and subsequent degradation. FOXM1 serves as another substrate for ubiquitination by STUB1. Additionally, STUB1 facilitates the translocation of FOXM1 from the nucleus to the cytoplasm, thereby reducing FOXM1’s binding to the TOP2A promoter. As a transcription factor for TOP2A, FOXM1 plays a crucial role in regulating its expression; thus, STUB1 downregulates TOP2A expression at the transcriptional level via FOXM1. Consequently, STUB1 modulates TOP2A levels through a dual mechanism involving both ubiquitination and FOXM1-mediated transcriptional repression. In breast cancer cells, this regulatory pathway enables STUB1 to inhibit tumor growth while enhancing sensitivity to doxorubicin

TOP2A is crucial for maintaining genomic stability, and is an important target for doxorubicin, etoposide, and other chemotherapy drugs. Abnormally elevated expression and catalytic activity of TOP2A have been noted in drug-resistant cancer cells, including breast cancer cells, which promote malignant progression of cancer and drug resistance to chemotherapy [21–23]. Therefore, TOP2A is a key mediator of chemoresistance in cancer cells. However, the regulation of TOP2A expression and activity remains largely unknown. In the present study, we report the regulation of TOP2A by STUB1 at both transcriptional and post-translational levels. STUB1 was identified as a key E3 ligase that regulates the stability of TOP2A. A series of experiments, including co-immunoprecipitation, GST pull-down, in vivo and in vitro ubiquitination, and CHX pulse-chase assays, demonstrated that TOP2A is a direct substrate of STUB1. STUB1 directly binds TOP2A and downregulates its expression at the post-translational level by promoting its ubiquitination and degradation. In addition, STUB1 downregulates the levels of the transcription factor FOXM1, thereby inhibiting transcription of TOP2A, whose promoter has recognition sites for this factor. This is an indirect pathway through which STUB1 regulates TOP2A at the transcriptional level. These results present a novel regulatory mechanism (Fig. 9). More importantly, the negative regulation of TOP2A by STUB1 may have significant clinical implications.

We evaluated the potential clinical significance of the negative regulation of TOP2A by STUB1 in breast cancer cells, xenograft models, and patients with breast cancer. In breast cancer cells, STUB1 inhibited the catalytic activity of TOP2A, suppressed cancer cell proliferation, and enhanced the susceptibility of these cells to apoptosis and cell-cycle arrest induced by doxorubicin. In the xenograft model of breast cancer, STUB1 also inhibited the growth of xenograft tumors and enhanced their sensitivity to doxorubicin. These effects were dependent on the inhibition of TOP2A expression. In patients with breast cancer that underwent EC-T neoadjuvant chemotherapy, those with elevated STUB1 expression and low TOP2A expression had significantly higher pCR rates. Univariate and multivariate analyses indicated that STUB1 and TOP2A were independent factors in predicting pCR. Therefore, increased expression levels of STUB1 in cancer cells can not only inhibit the expression and activity of TOP2A to curb malignant progression but also reduce the occurrence of chemotherapy resistance and thus contribute to achieving better therapeutic effects. The elevated expression of TOP2A across multiple cancer types suggests that the regulatory mechanism described herein, along with their corresponding clinical implications, may be applicable to a wider range of malignancies; however, further experimental validation is necessary. In addition, this study cohort is confined to a single institution and has a limited sample size. Therefore, it will be valuable to corroborate these findings in future multicenter studies.

In addition to TOP2A, FOXM1 was identified as another ubiquitination substrate for STUB1 in this study. FOXM1 is an oncogenic transcription factor that regulates the cell cycle, and is highly associated with drug resistance during cancer treatment. Recent research indicates that FOXM1 confers chemoresistance to various types of cancers by regulating multiple signaling cascades [24–26]. Therefore, FOXM1 is an important molecule for tumor-targeted therapy and related drug research. Similar to TOP2A, its regulatory mechanisms have received considerable attention. Our study reveals for the first time that FOXM1 functions as a transcription factor for TOP2A and that its stability is regulated by ubiquitination mediated by STUB1. In addition to TOP2A, there are multiple target molecules regulated by FOXM1, involved in tumorigenesis, tumor progression, and drug resistance [27–29]. Therefore, STUB1 may have a broader role in regulating these processes via FOXM1, which warrants further exploration and research.

Notably, our study findings revealed the translocation of TOP2A and FOXM1 from the nucleus to the cytoplasm following transfection with a STUB1-containing vector. The presence of TOP2A within the nucleus is necessary for its function, as only those molecules located within the nucleus can access the DNA. Similarly, FOXM1 must be transported to the nucleus after its synthesis in the cytoplasm to function as a transcription factor. Therefore, even without causing changes in their expression levels, STUB1 can affect their function by altering their subcellular localization. Increasing evidence suggests that post-translational modifications such as ubiquitination, deubiquitination, and SUMOylation often alter the subcellular localization of target proteins [30–32]; however, the fundamental mechanisms involved on this continue to be inadequately understood. Our present results do not allow us to determine the specific mechanism by which STUB1 induces the cytoplasmic translocation of TOP2A and FOXM1. One possibility is that some ubiquitination sites in TOP2A and FOXM1 are located within their nuclear localization signal (NLS) sequences [33, 34], and ubiquitination may interfere with their nuclear localization directed by these sequences. However, further studies are required to test this hypothesis.

In summary, our study proposes a novel and important regulatory mechanism involving STUB1, FOXM1, and TOP2A. As an E3 ubiquitin ligase, STUB1 induces the ubiquitination and subsequent degradation of TOP2A and FOXM1. Furthermore, FOXM1 acts as a transcription factor for TOP2A, and STUB1 inhibits TOP2A transcription through FOXM1, thereby suppressing breast cancer proliferation and enhancing sensitivity to chemotherapy. Our results suggest that the expression level of STUB1 can serve as a predictor of responsiveness to chemotherapy. Moreover, increasing the expression level of STUB1 in cancer cells may help reduce the occurrence of chemotherapy resistance and thus improve outcomes in patients with cancer.

## Supplementary Information

Additional file 1.

Additional file 2.

## Data Availability

No datasets were generated or analyzed during the current study.

## Abbreviations

TOP2A: DNA topoisomerase II alpha
STUB1: STIP1 homology and U-box-containing protein 1
GST: Glutathione S-transferases
CHX: Cycloheximide
CHIP: Chromatin immunoprecipitation
FOXM1: Forkhead box M1
pCR: Pathologic complete response
HER2: Human epidermal growth factor receptor 2
qPCR: Quantitative RT-PCR
