# Supplementary material for: STUB1 downregulates TOP2A through a dual mechanism of ubiquitination and FOXM1-mediated transcription repression, suppressing breast cancer growth and enhancing sensitivity to chemotherapy
Source: Cell Mol Biol Lett. 2026 Mar 18;31:65. doi: 10.1186/s11658-026-00902-2 (PMC13122884; doi:10.1186/s11658-026-00902-2)

Figure 1A

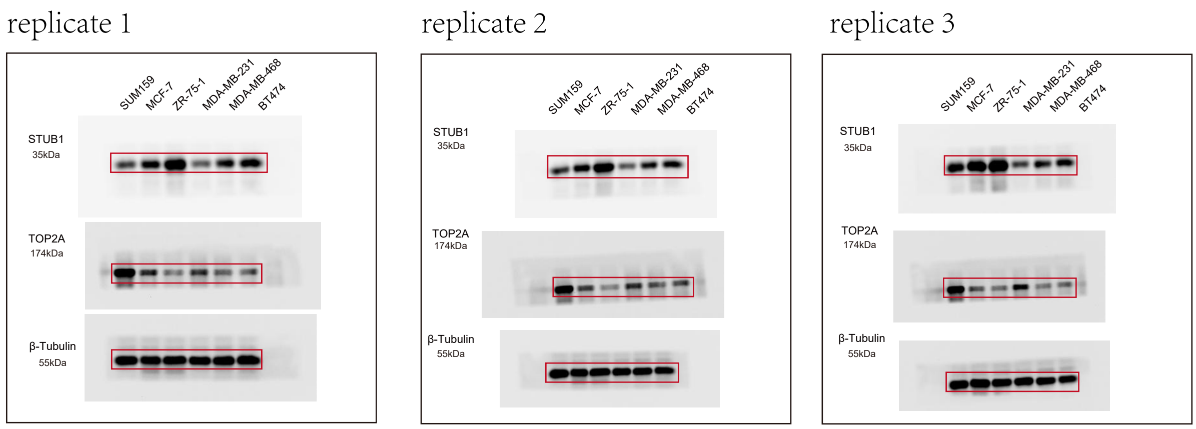

Figure 1B

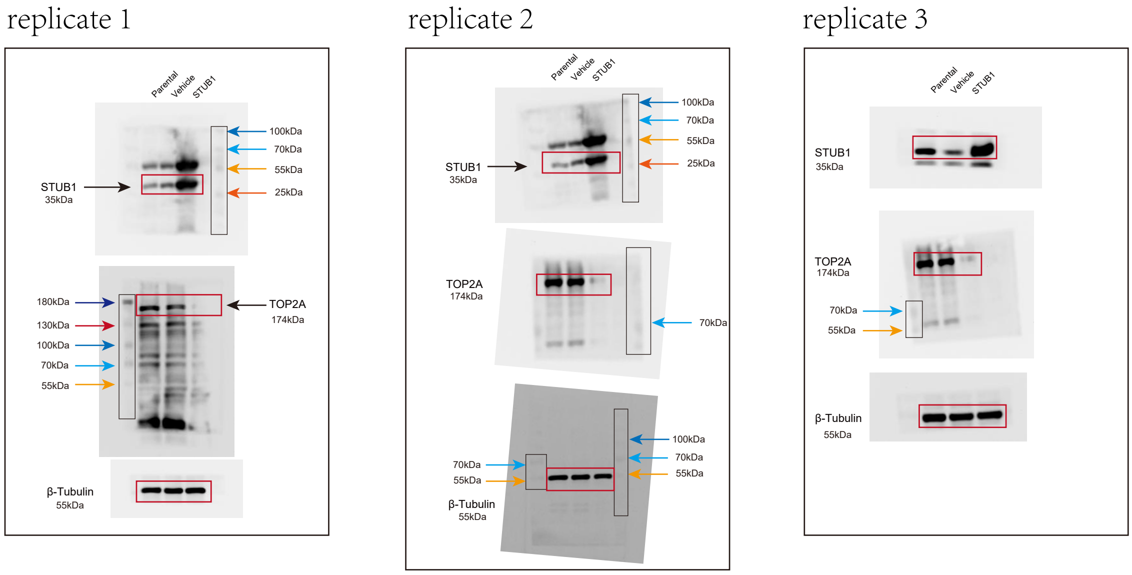

Figure 1C

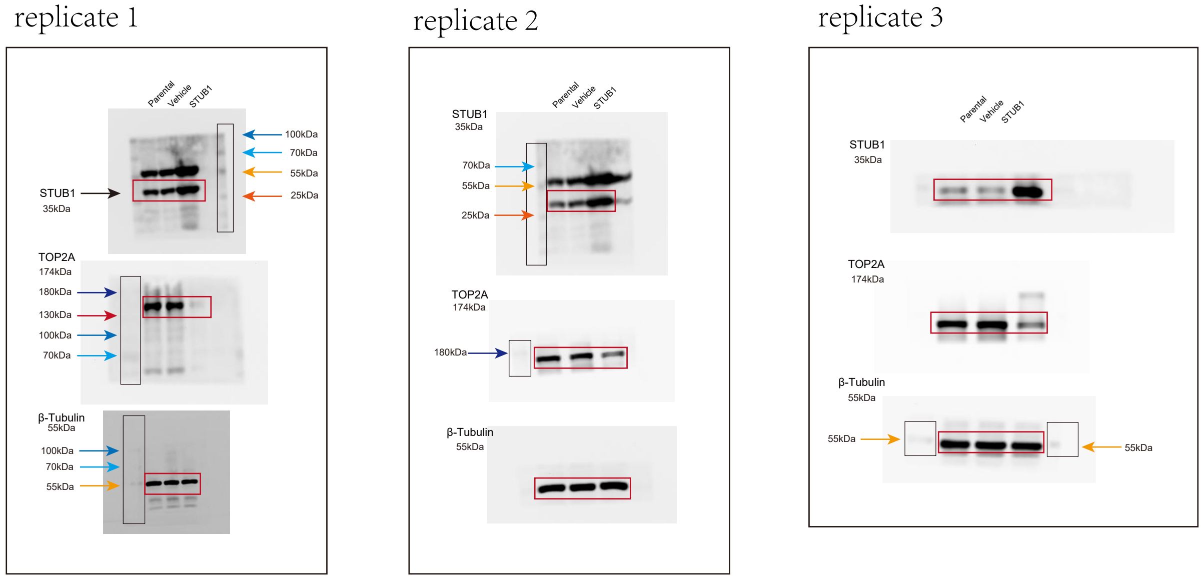

Figure 1D

replicate 1

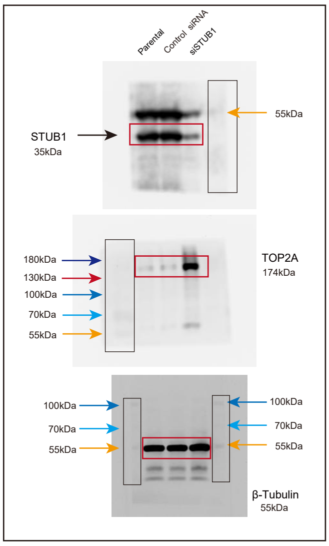

replicate 2

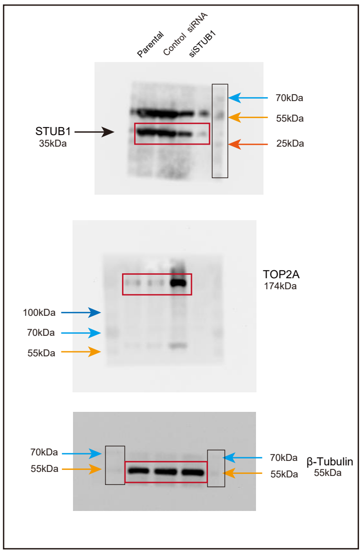

replicate 3

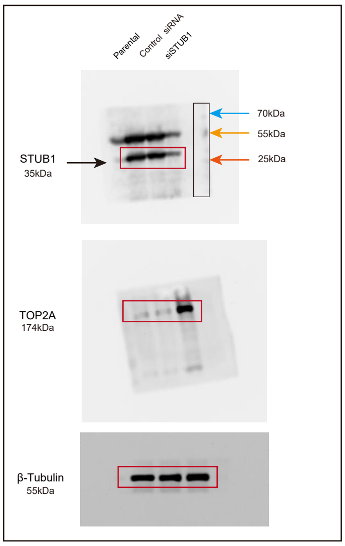

Figure 1E

replicate 1

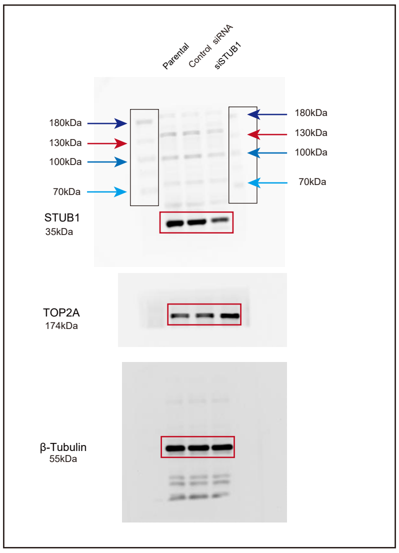

replicate 2

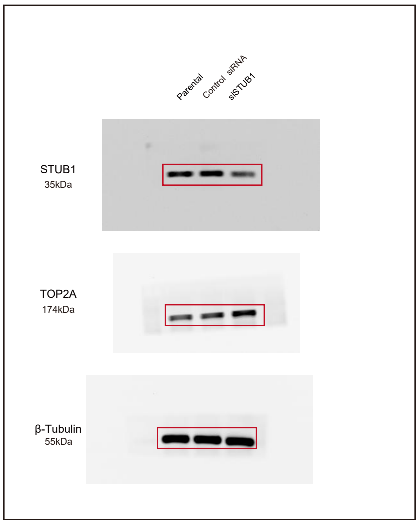

replicate 3

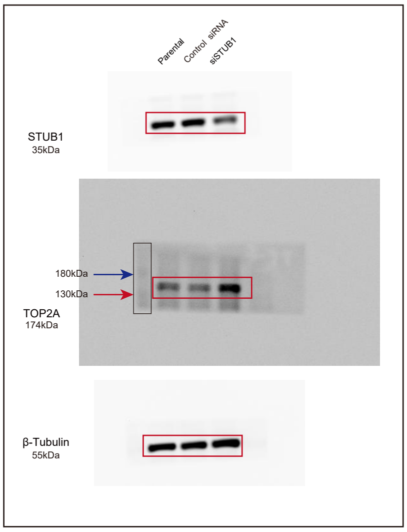

Figure 1F

replicate 1

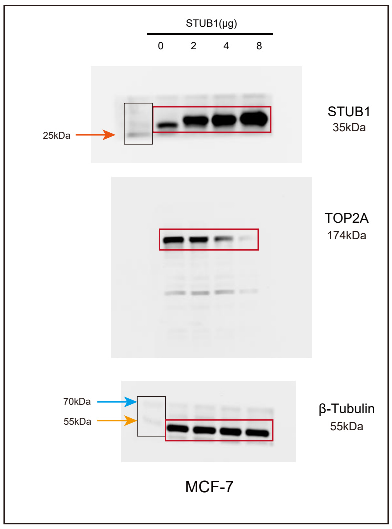

replicate 2

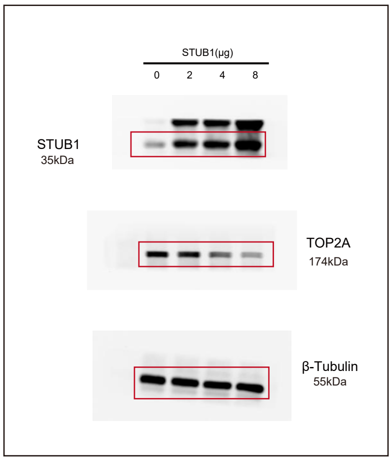

replicate 3

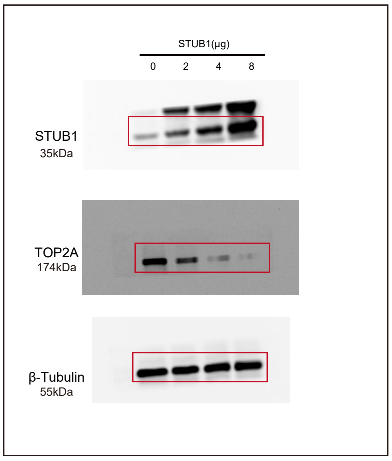

Figure 2A

IP:TOP2A replicate 1

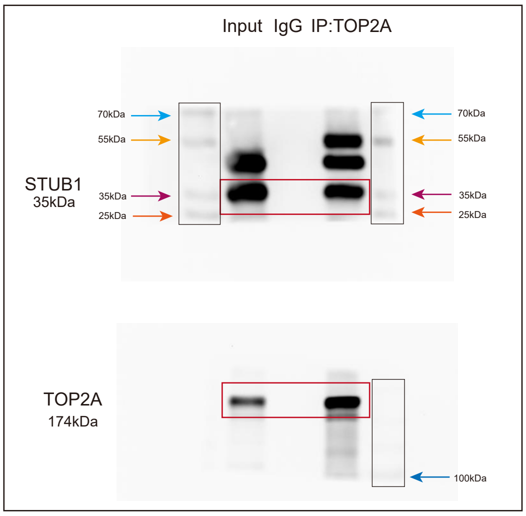

replicate 2

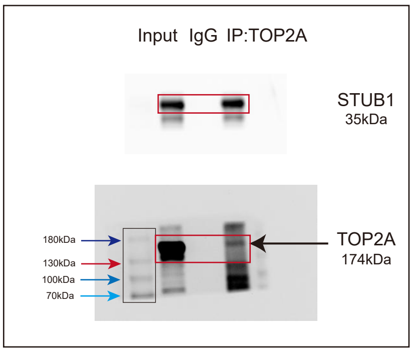

replicate 3

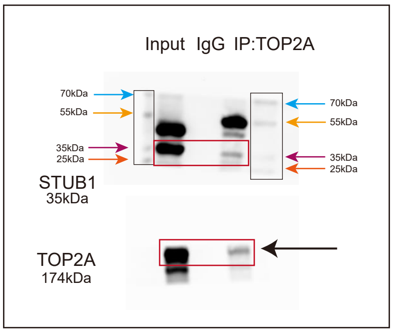

IP:STUB1 replicate 1

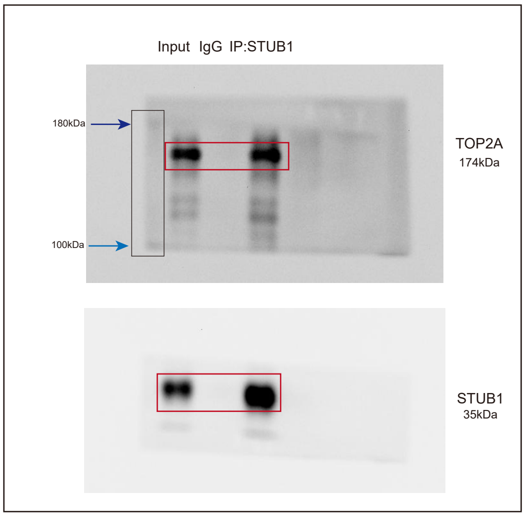

replicate 2

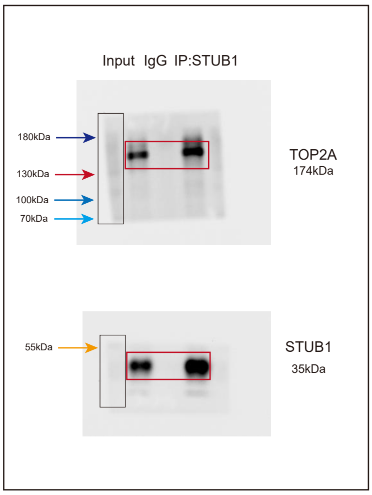

replicate 3

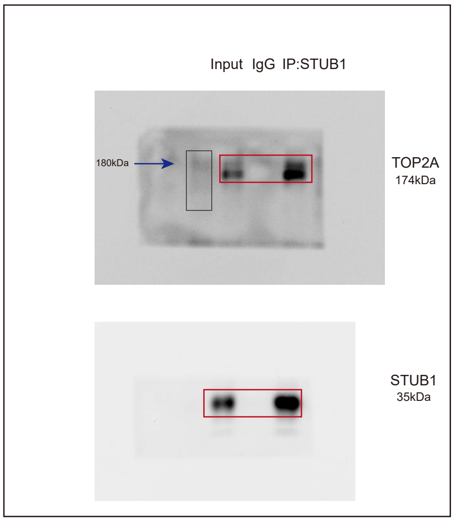

Figure 2C

replicate 1

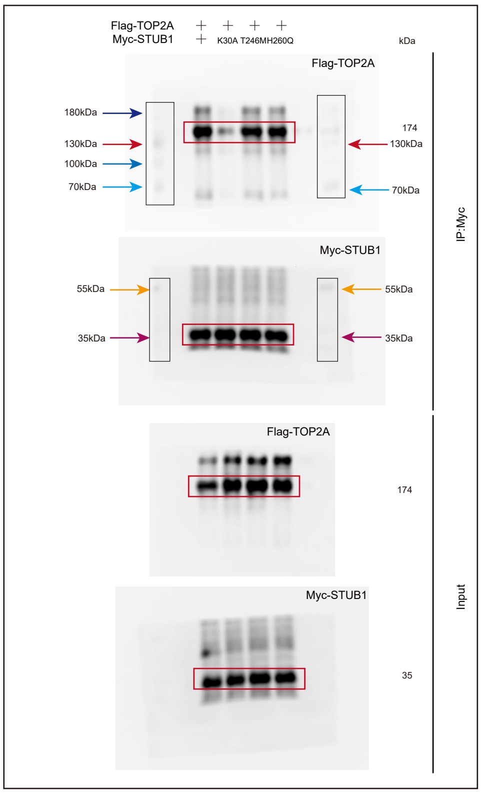

replicate 2

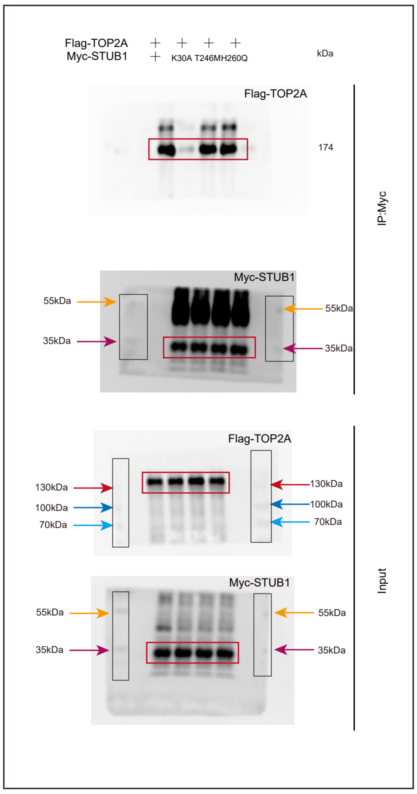

replicate 3

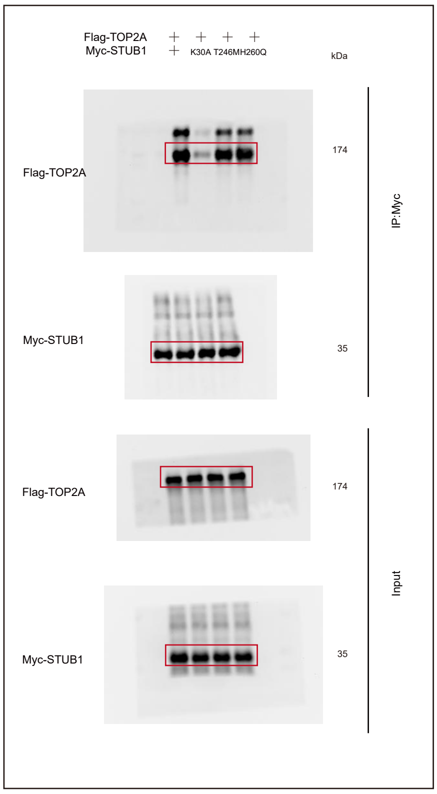

Figure 2E

replicate 1

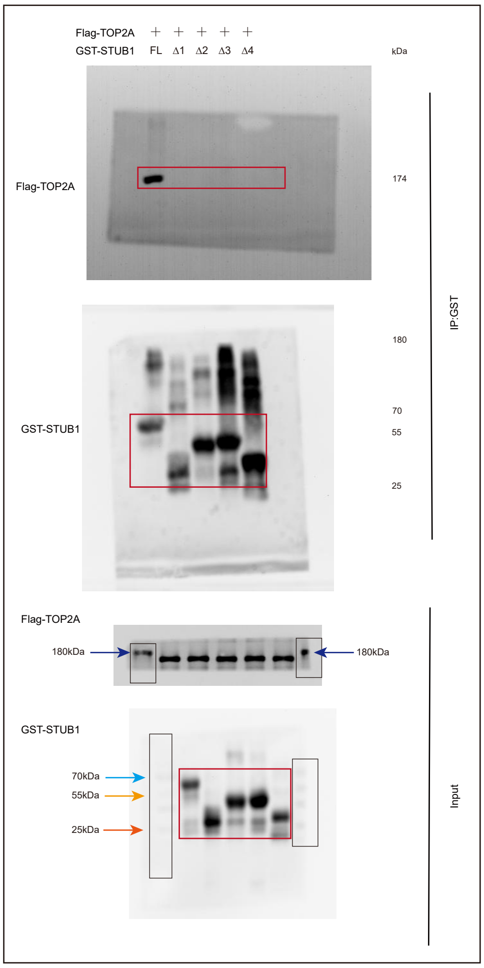

replicate 2

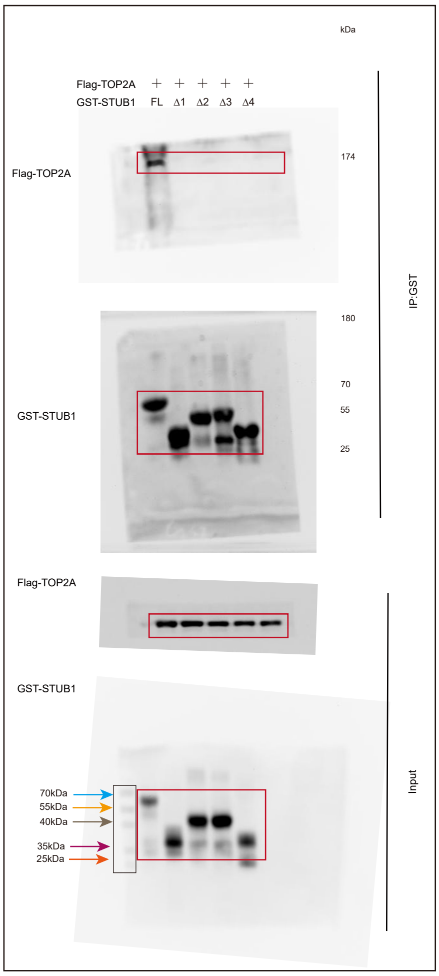

replicate 3

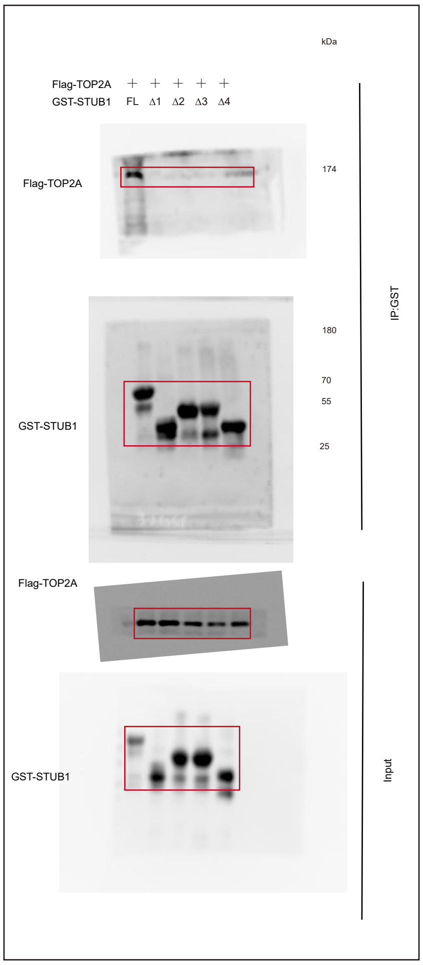

Figure 2G

replicate 1

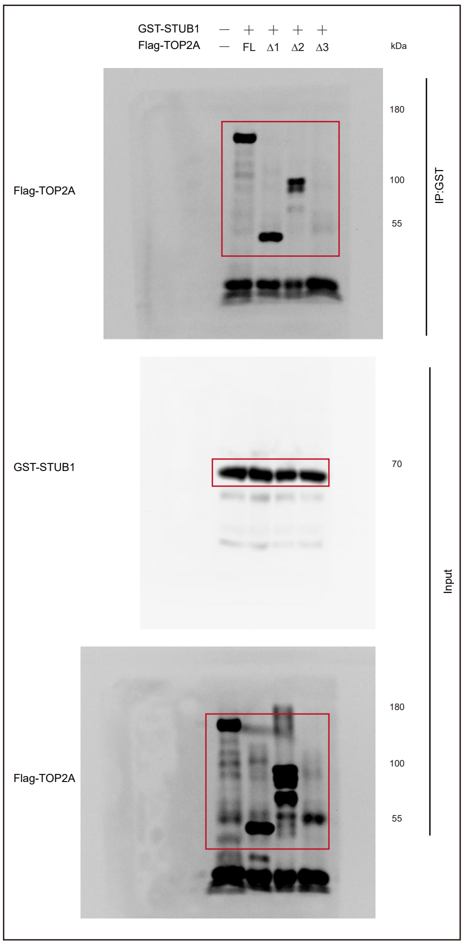

replicate 2

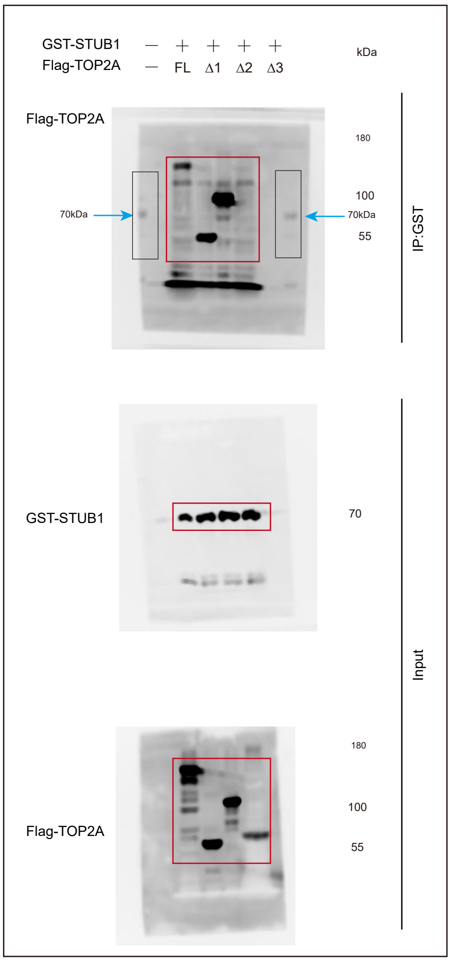

replicate 3

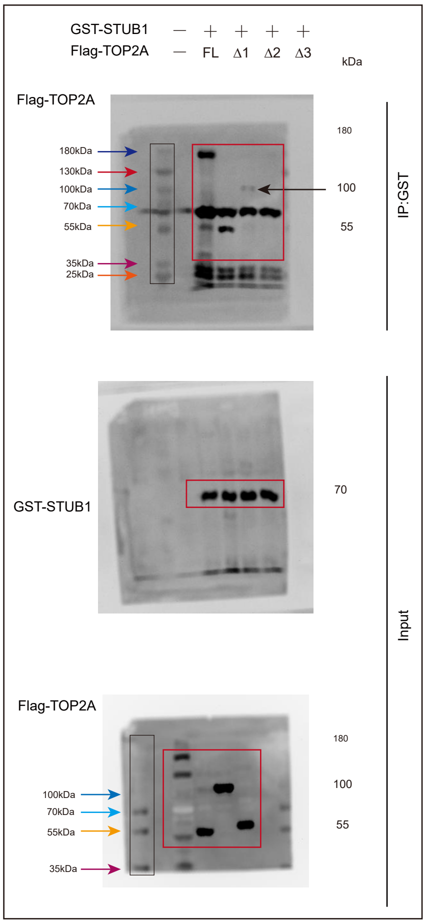

Figure 3A

replicate 1

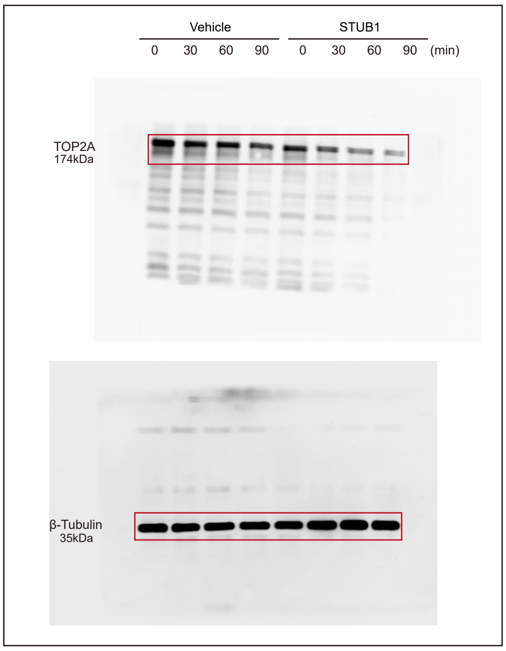

replicate 2

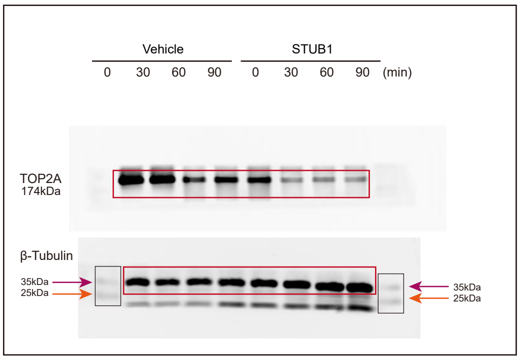

replicate 3

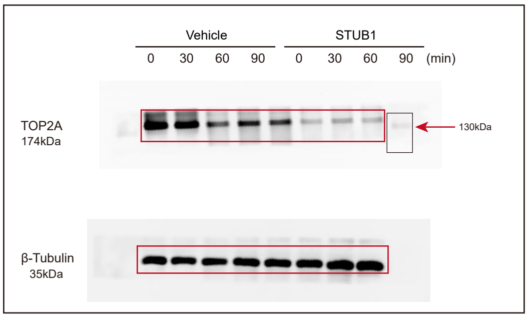

Figure 3B

replicate 1

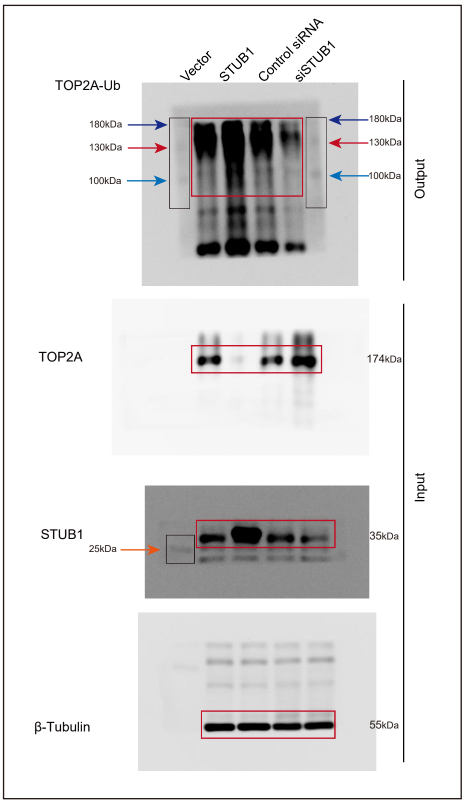

replicate 2

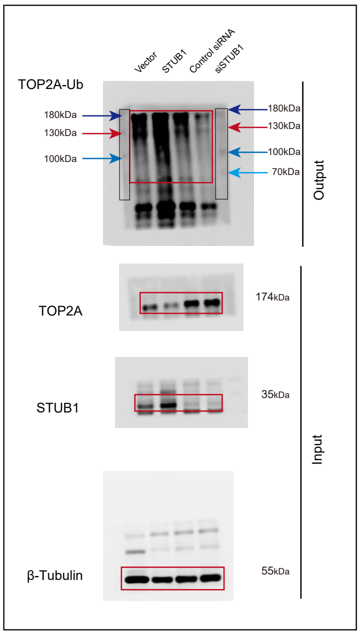

replicate 3

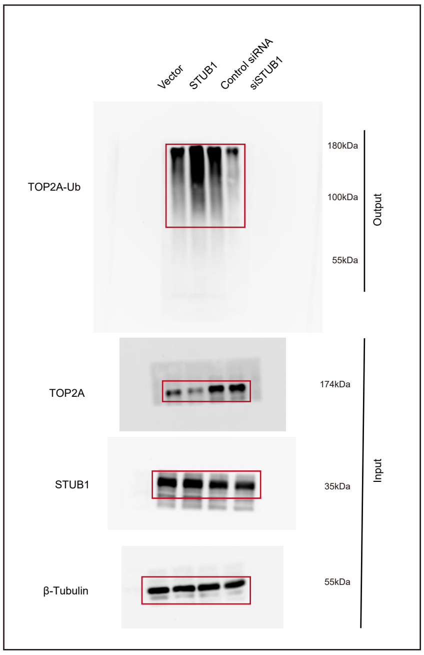

Figure 3C

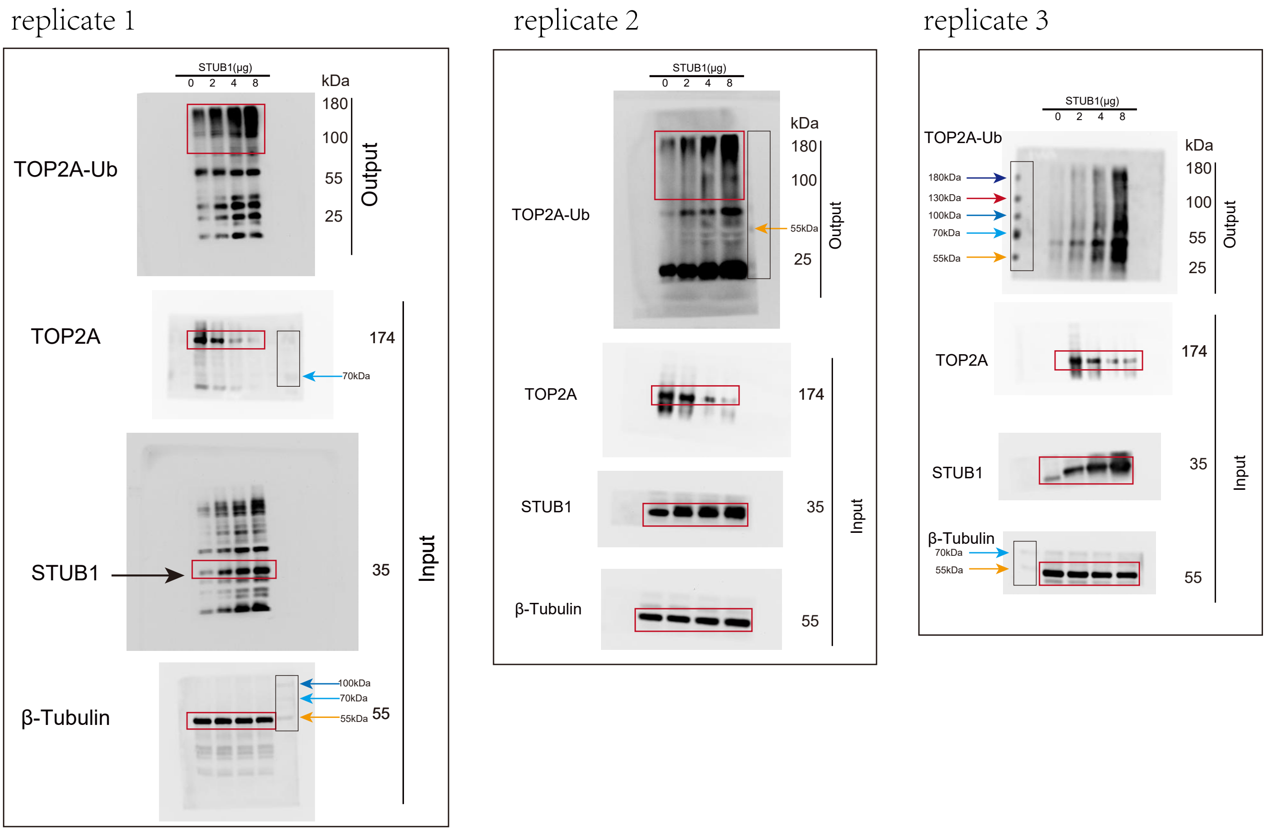

Figure 3D

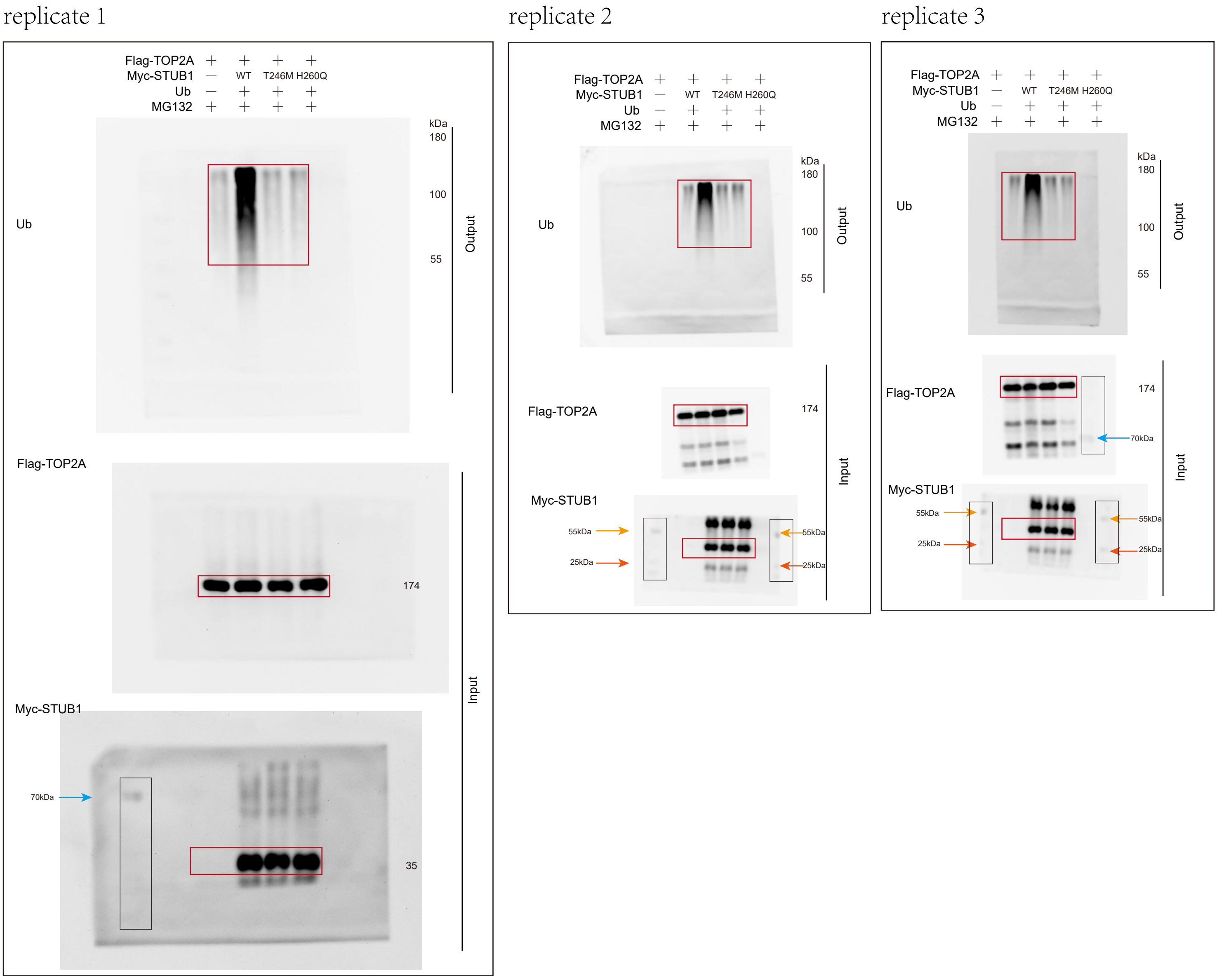

Figure 3E

replicate 1

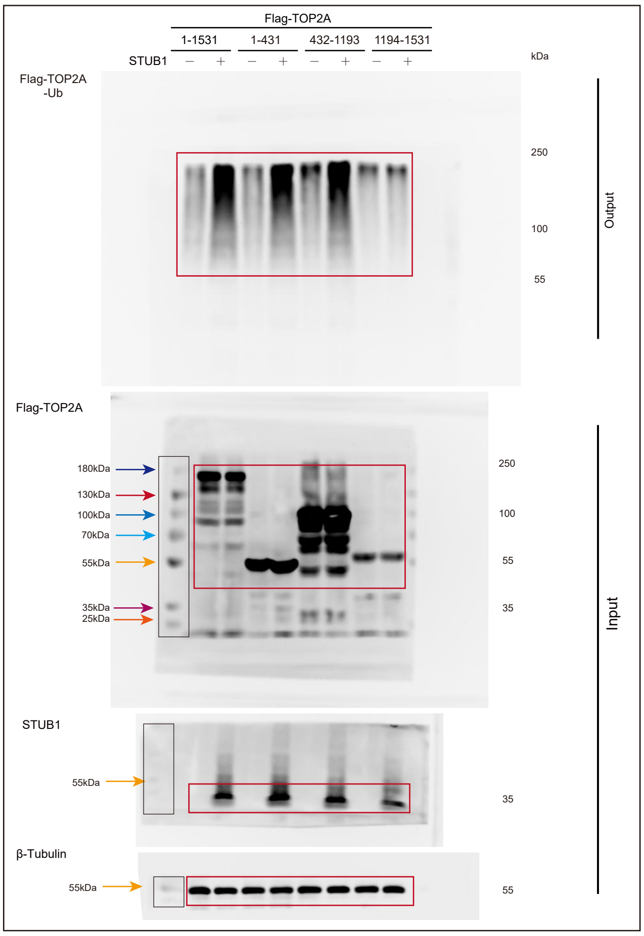

replicate 2

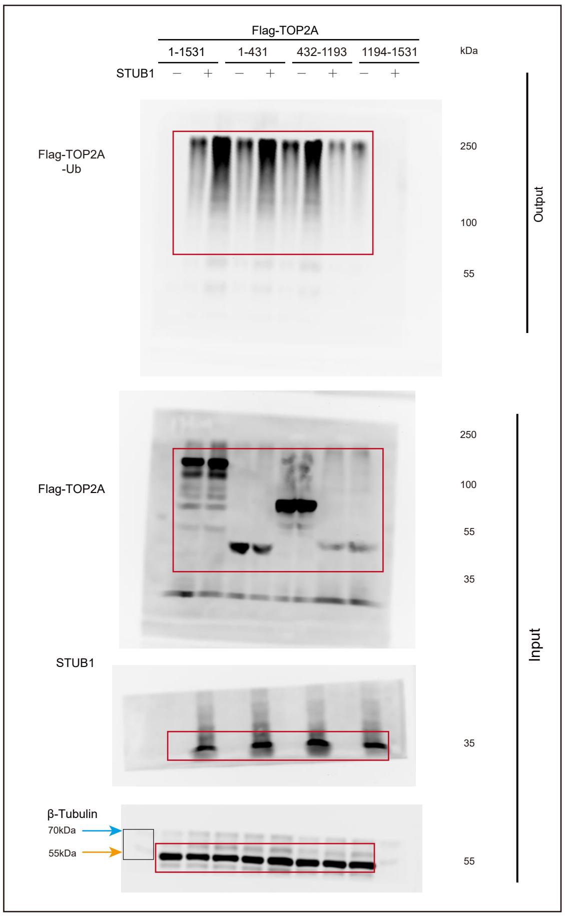

replicate 3

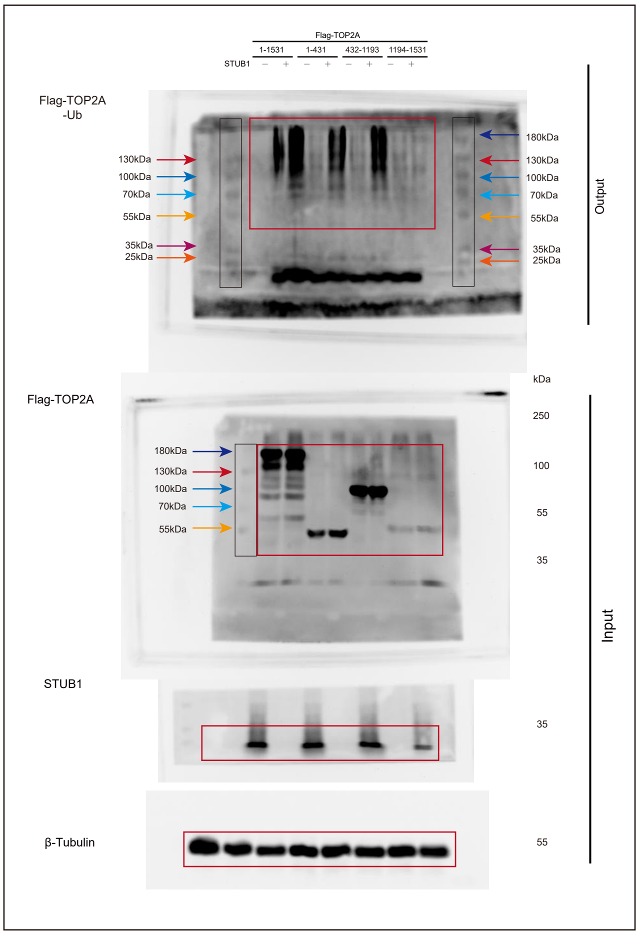

Figure 3F

replicate 1

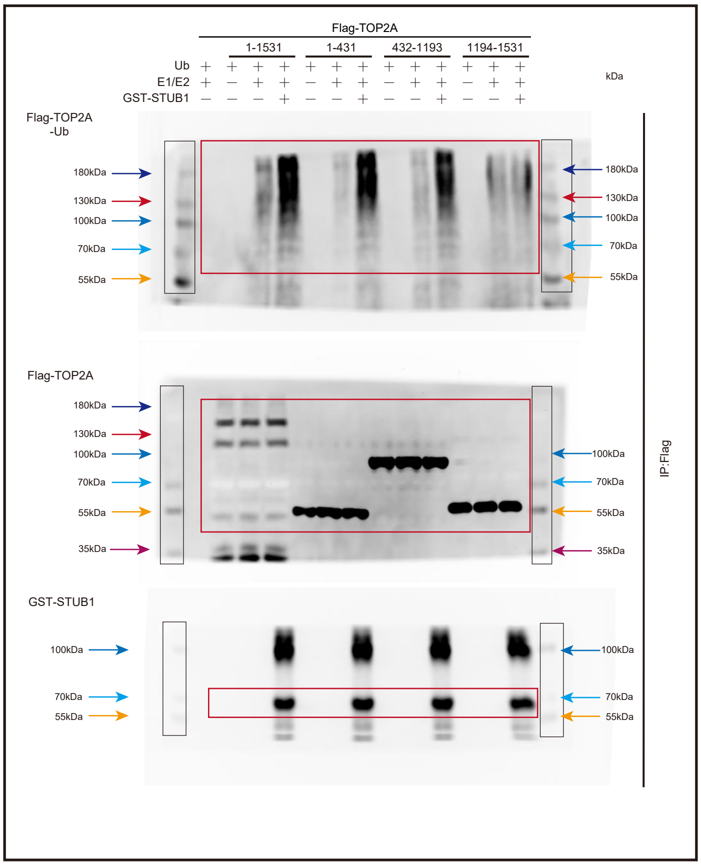

replicate 2

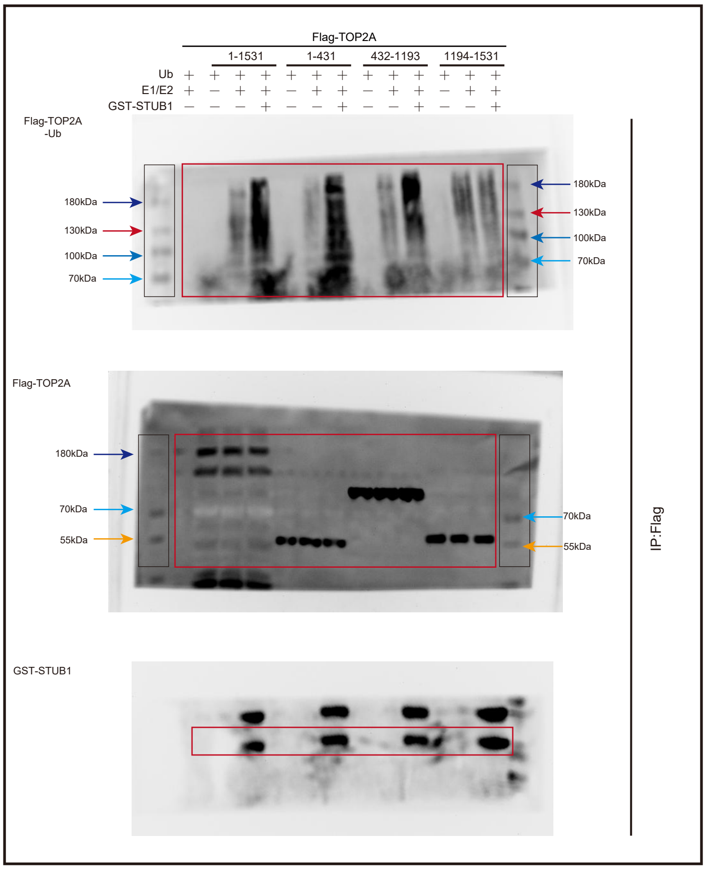

replicate 3

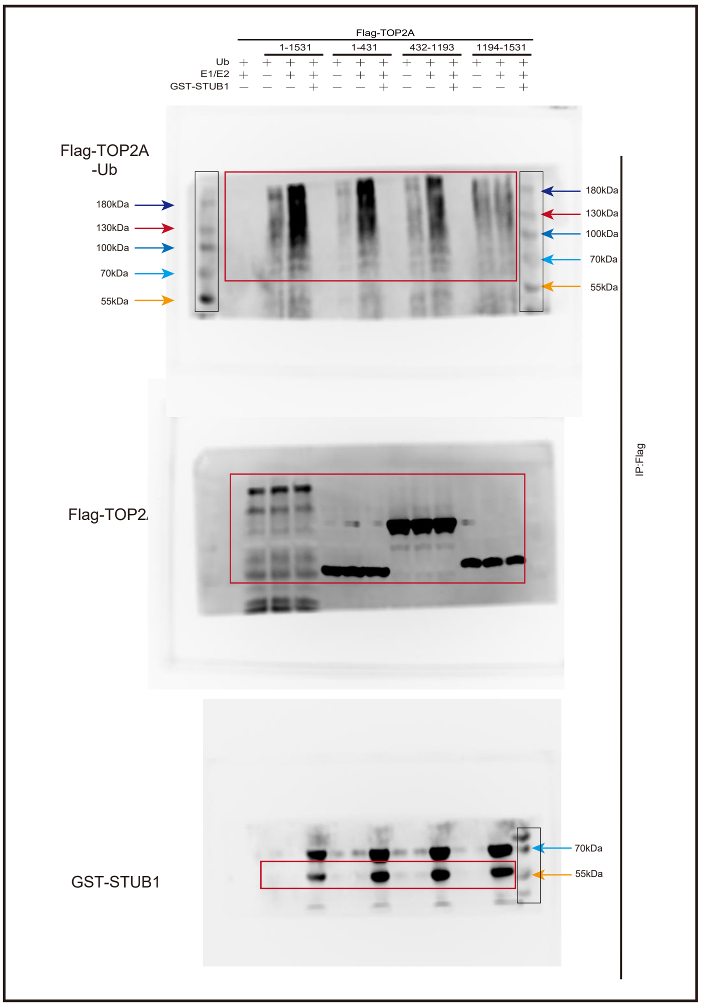

Figure 4E

replicate 1

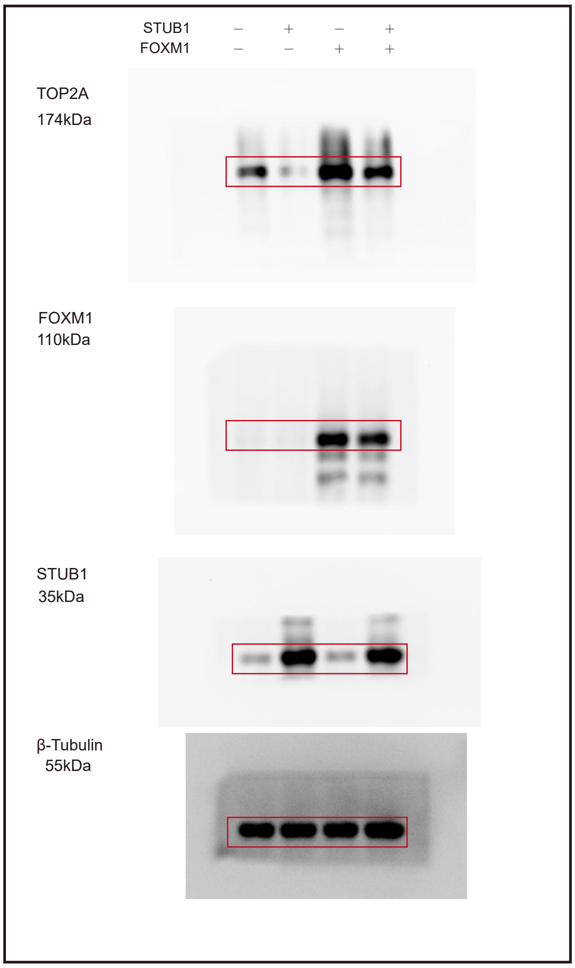

replicate 2

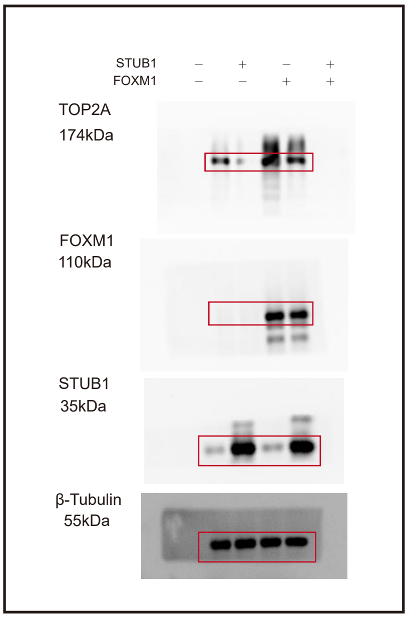

replicate 3

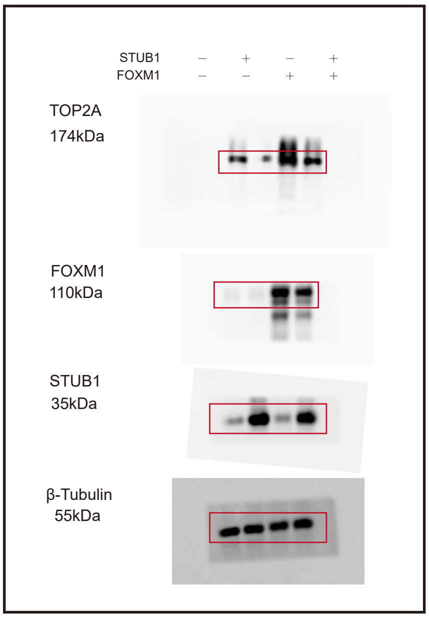

Figure 5A

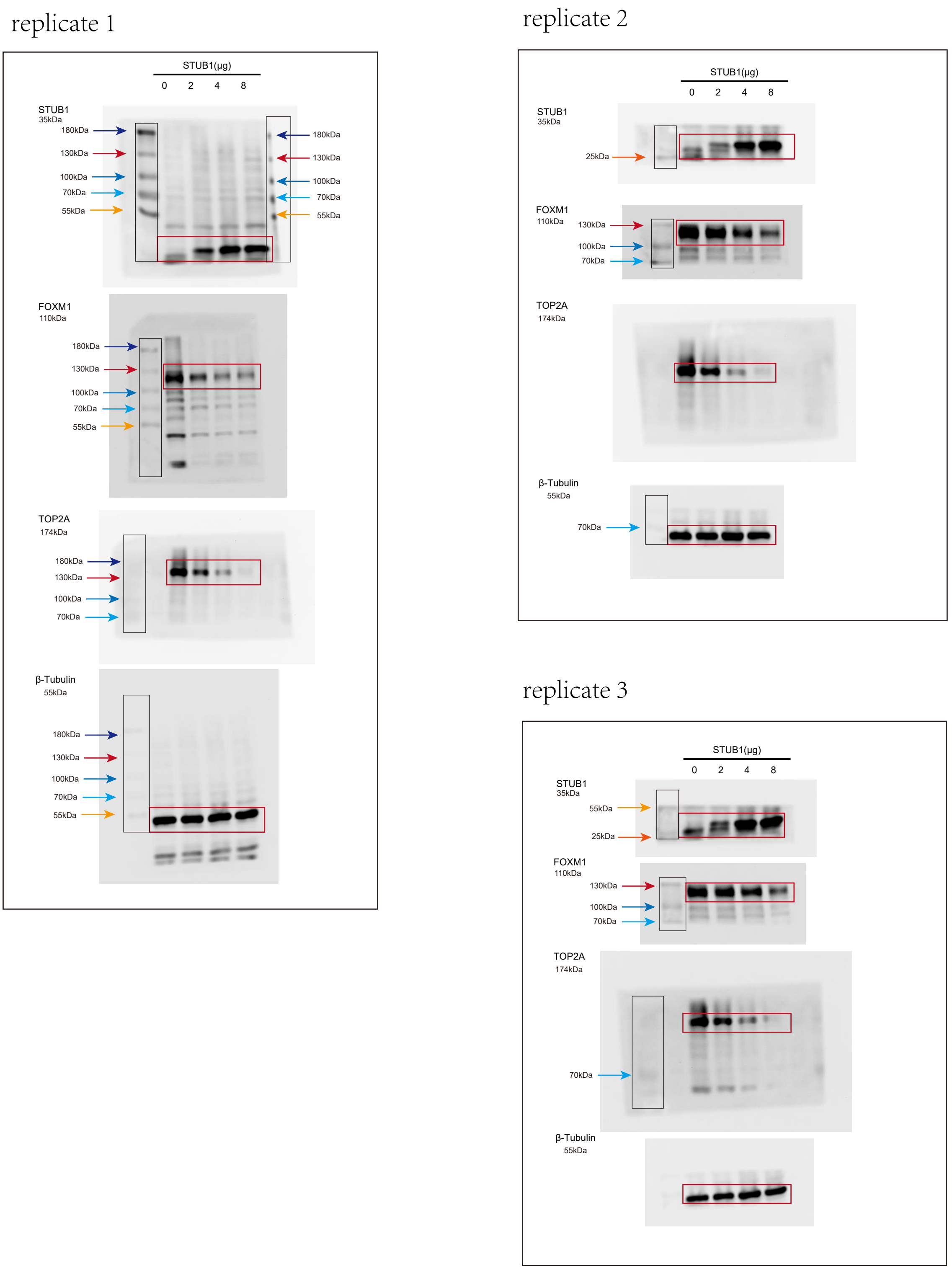

replicate 1
STUB1(μg)
0 2 4 8
STUB1
35kDa
180kDa
130kDa
100kDa
70kDa
55kDa
FOXM1
110kDa
TOP2A
174kDa
β-Tubulin
55kDa
replicate 2
STUB1(μg)
0 2 4 8
STUB1
35kDa
25kDa
FOXM1
110kDa
130kDa
100kDa
70kDa
TOP2A
174kDa
β-Tubulin
55kDa
70kDa
replicate 3
STUB1(μg)
0 2 4 8
STUB1
35kDa
55kDa
25kDa
FOXM1
110kDa
130kDa
100kDa
70kDa
TOP2A
174kDa
70kDa
β-Tubulin
55kDa

Figure 5B

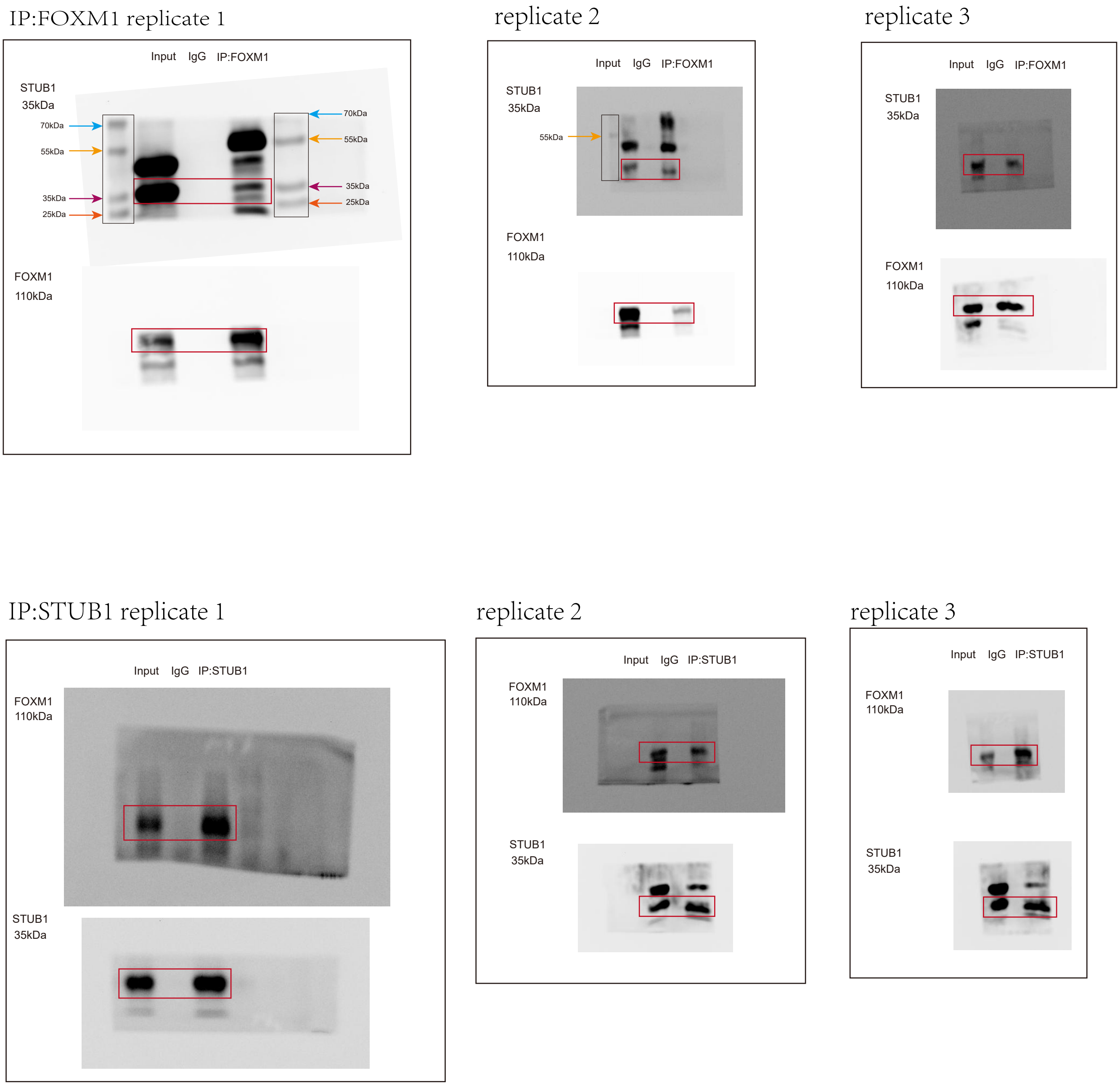
IP:FOXM1 replicate 1
Input IgG IP:FOXM1
STUB1
35kDa
70kDa
55kDa
35kDa
25kDa
70kDa
55kDa
35kDa
25kDa
FOXM1
110kDa
replicate 2
Input IgG IP:FOXM1
STUB1
35kDa
55kDa
FOXM1
110kDa
replicate 3
Input IgG IP:FOXM1
STUB1
35kDa
FOXM1
110kDa
IP:STUB1 replicate 1
Input IgG IP:STUB1
FOXM1
110kDa
STUB1
35kDa
replicate 2
Input IgG IP:STUB1
FOXM1
110kDa
STUB1
35kDa
replicate 3
Input IgG IP:STUB1
FOXM1
110kDa
STUB1
35kDa

Figure 5C

replicate 1

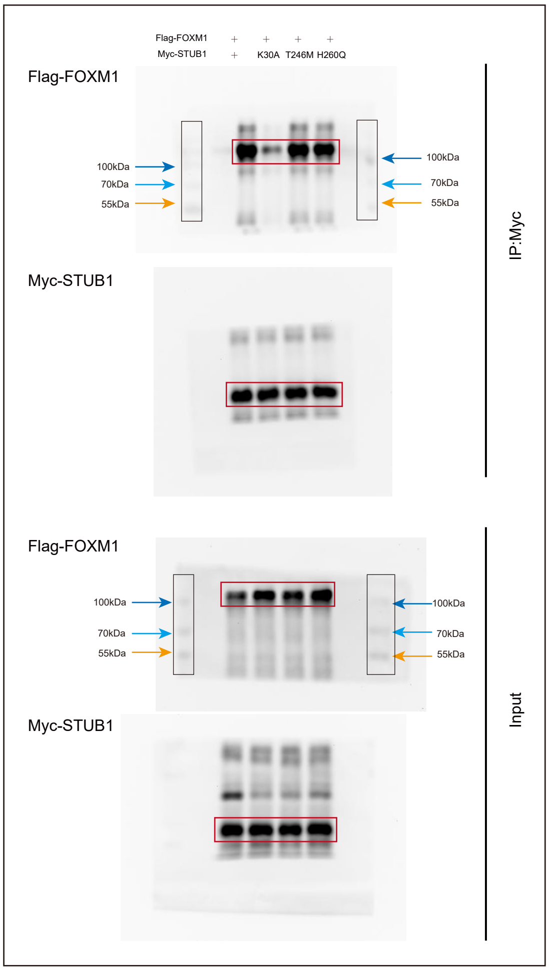

replicate 2

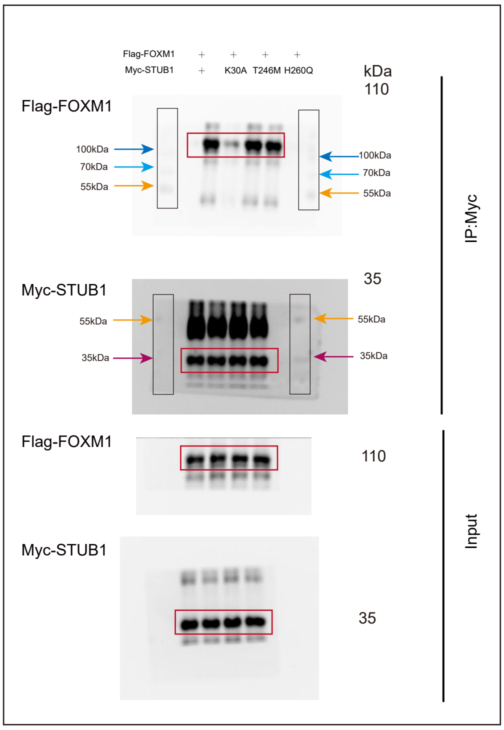

replicate 3

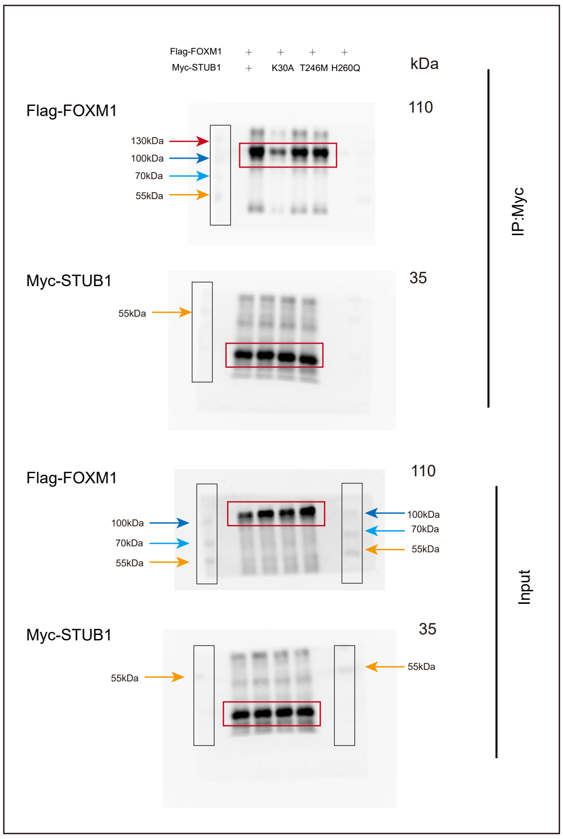

Figure 5E

replicate 1

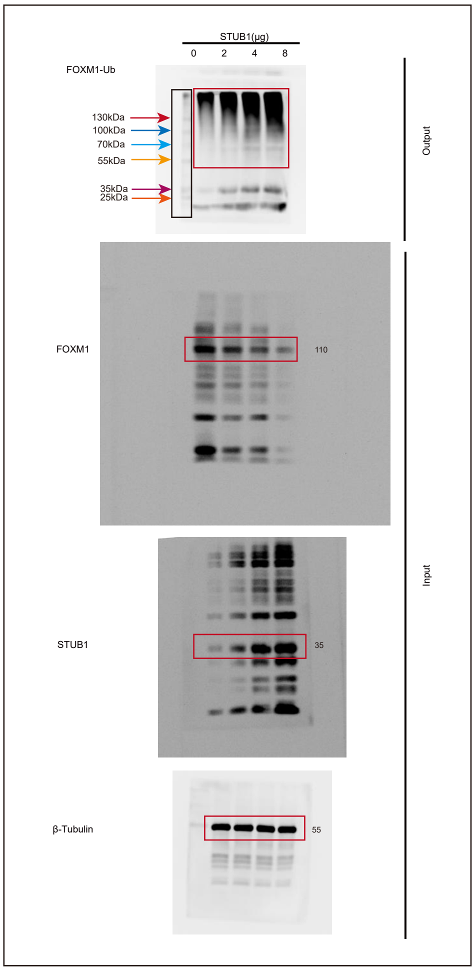

replicate 2

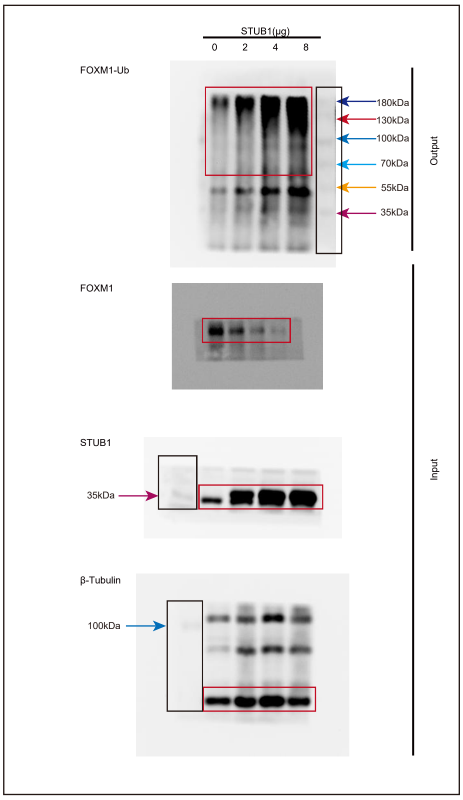

replicate 3

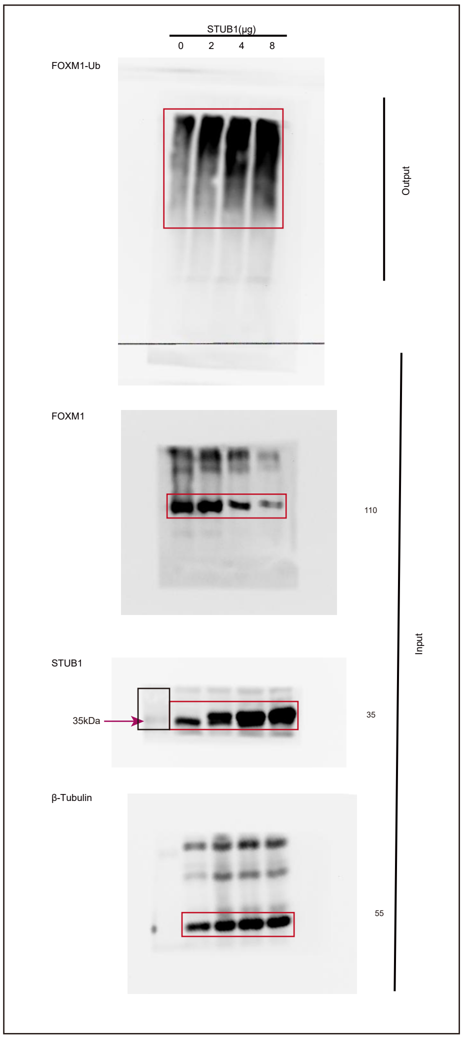

Figure 5F

replicate 1

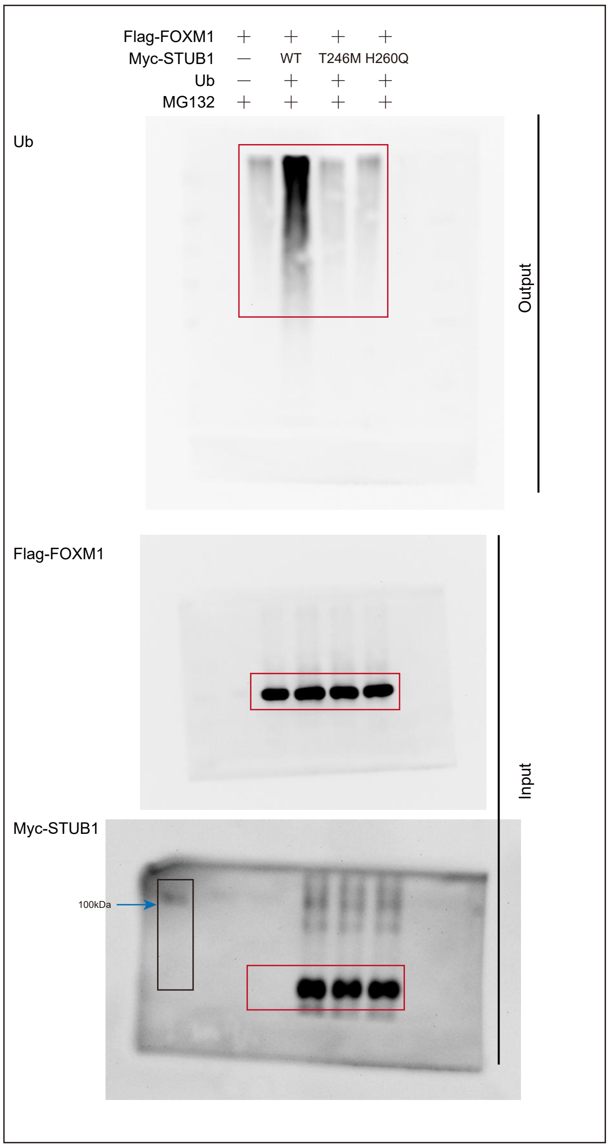

replicate 2

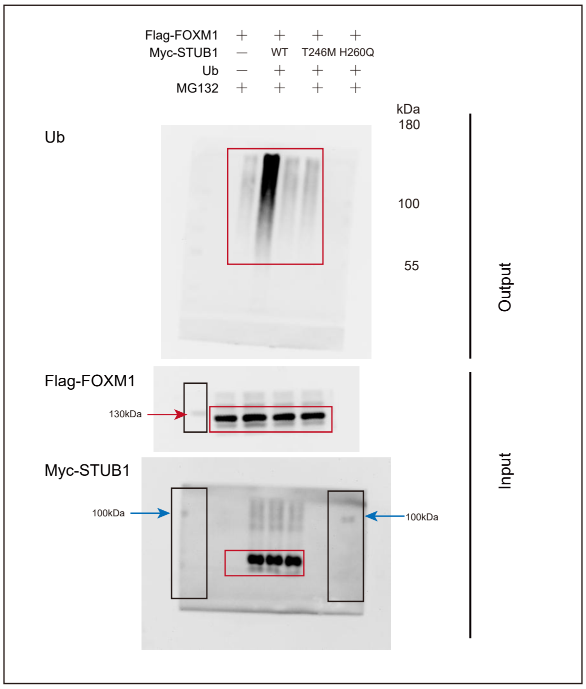

replicate 3

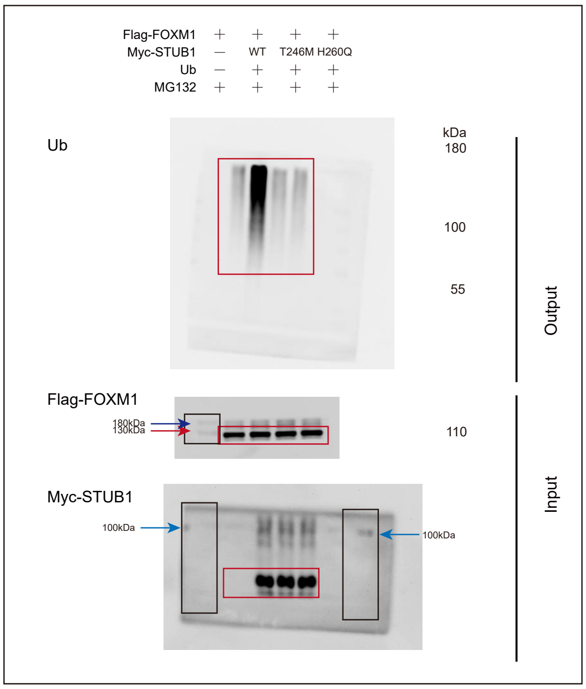

Figure 5G

replicate 1

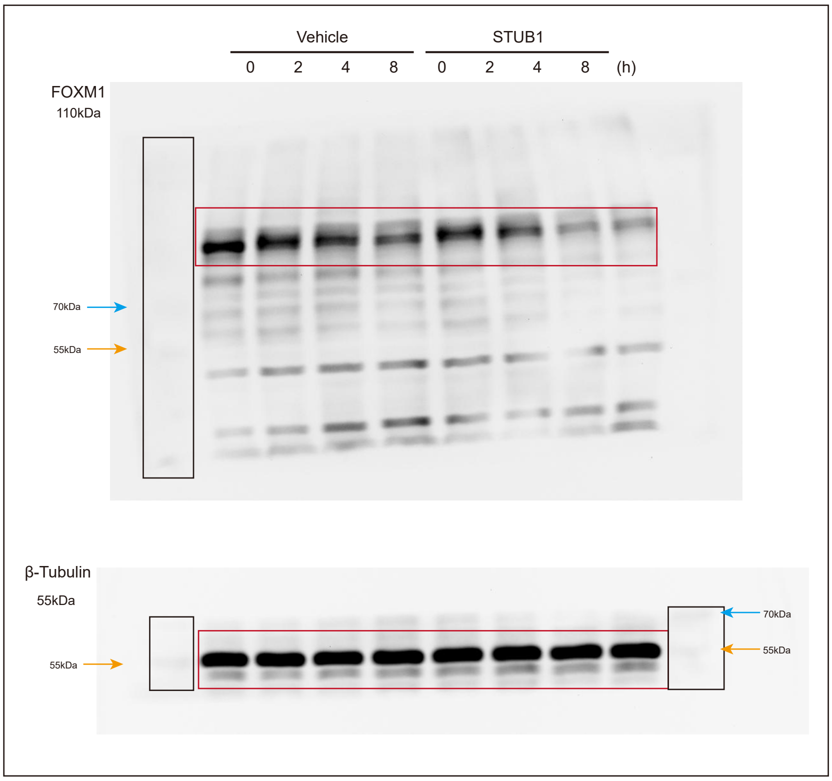

replicate 2

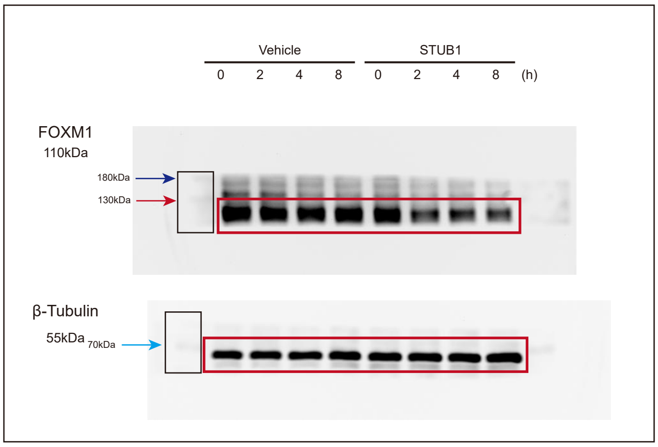

replicate 3

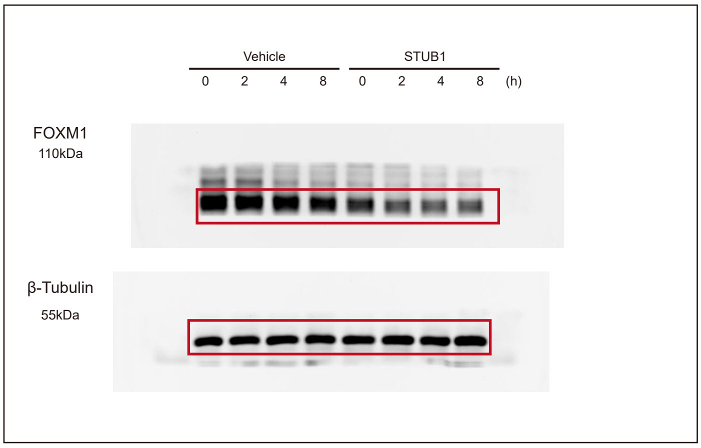

Figure 7E

replicate 1

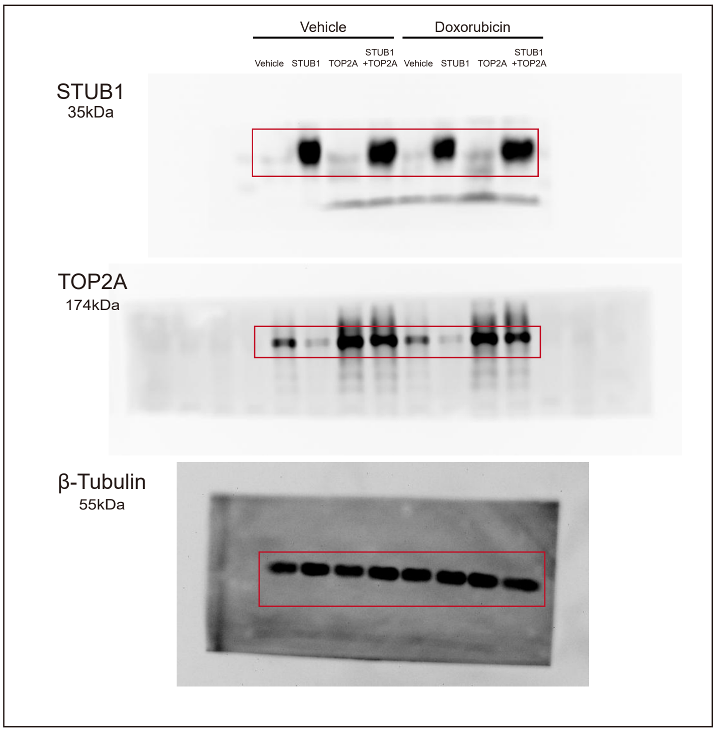

replicate 2

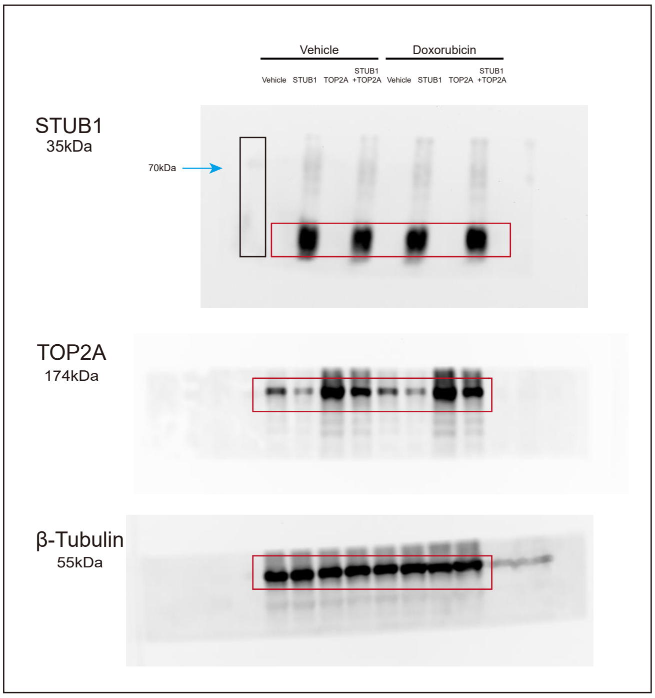

replicate 3

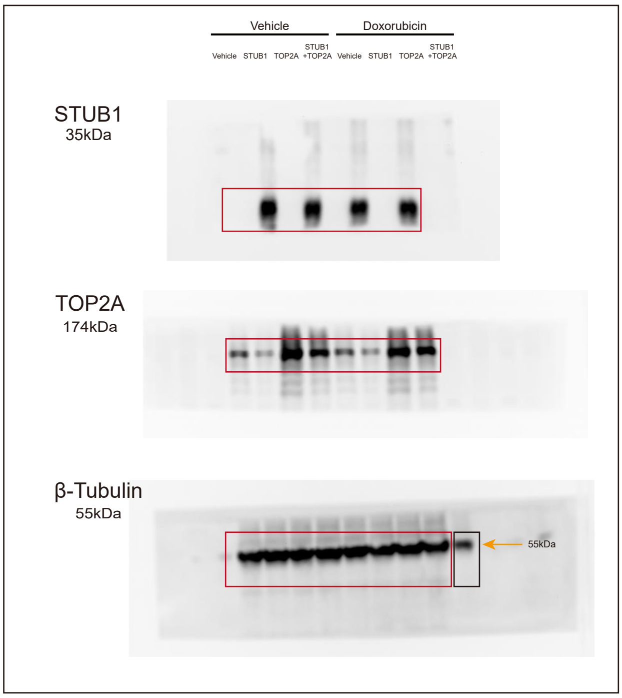

Supplement: Supplementary file 2 — Additional file 2. [file 11658_2026_902_MOESM2_ESM.pdf]
